# Cost-effectiveness of transdiagnostic group cognitive behavioural therapy versus group relaxation therapy for emotional disorders in primary care (PsicAP-Costs2): Protocol for a multicentre randomised controlled trial

**DOI:** 10.1371/journal.pone.0283104

**Published:** 2023-03-16

**Authors:** César González-Blanch, Sara Barrio-Martínez, Amador Priede, Sandra Martínez-Gómez, Saioa Pérez-García-Abad, María Miras-Aguilar, José Ruiz-Gutiérrez, Roger Muñoz-Navarro, Paloma Ruiz-Rodríguez, Leonardo A. Medrano, Maider Prieto-Vila, María Carpallo-González, Ángel Aguilera-Martín, Mario Gálvez-Lara, Fátima Cuadrado, Eliana Moreno, Francisco García-Torres, José F. Venceslá, Jorge Corpas, Francisco J. Jurado-González, Juan A. Moriana, Antonio Cano-Vindel

**Affiliations:** 1 Mental Health Centre, Marqués de Valdecilla University Hospital—IDIVAL, Santander, Spain; 2 Faculty of Health Sciences, Universidad Europea del Atlántico, Santander, Spain; 3 Faculty of Psychology, Complutense University of Madrid, Madrid, Spain; 4 Valdecilla Biomedical Research Institute (IDIVAL), Santander, Spain; 5 Mental Health Centre, Hospital de Laredo, Laredo, Spain; 6 Resident of Clinical Psychology, Marqués de Valdecilla University Hospital, Santander, Spain; 7 Department of Personality, Assessment and Psychological Treatments, Faculty of Psychology, University of Valencia, Valencia, Spain; 8 Castilla La Nueva Primary Care Centre, Health Service of Madrid, Fuenlabrada, Madrid, Spain; 9 Pontificia Universidad Católica Madre y Maestra, Santiago De Los Caballeros, Dominican Republic; 10 Department of Psychology, Faculty of Education Sciences, University of Cordoba, Cordoba, Spain; 11 Maimónides Biomedical Research Institute of Cordoba, Reina Sofía University Hospital, Cordoba, Spain; Institute of Biomedical and Health Research in Malaga (IBIMA), SPAIN

## Abstract

Several randomised controlled trials (RCT) have demonstrated the superiority of transdiagnostic group cognitive-behavioural therapy (TD-CBT) to treatment as usual (TAU) for emotional disorders in primary care. To date, however, no RCTs have been conducted to compare TD-CBT to another active intervention in this setting. Our aim is to conduct a single-blind RCT to compare group TD-CBT plus TAU to progressive muscle relaxation (PMR) plus TAU in adults (age 18 to 65 years) with a suspected emotional disorder. We expect that TD-CBT + TAU will be more cost-effective than TAU + PMR, and that these gains will be maintained at the 12-month follow-up. Seven therapy sessions (1.5 hours each) will be offered over a 24-week period. The study will be carried out at four primary care centres in Cantabria, Spain. The study will take a societal perspective. Psychological assessments will be made at three time points: baseline, post-treatment, and at 12-months. The following variables will be evaluated: clinical symptoms (anxiety, depression, and/or somatic); functioning; quality of life (QoL); cognitive-emotional factors (rumination, worry, attentional and interpretative biases, emotion regulation and meta-cognitive beliefs); and satisfaction with treatment. Data on health service use, medications, and sick days will be obtained from electronic medical records. Primary outcome measures will include: incremental cost-effectiveness ratios (ICER) and incremental cost-utility ratios (ICURs). Secondary outcome measures will include: clinical symptoms, QoL, functioning, and treatment satisfaction. Bootstrap sampling will be used to assess uncertainty of the results. Secondary moderation and mediation analyses will be conducted. Two questionnaires will be administered at sessions 1, 4, and 7 to assess therapeutic alliance and group satisfaction. If this trial is successful, widespread application of this cost-effective treatment could greatly improve access to psychological treatment for emotional disorders in the context of increasing demand for mental healthcare in primary care.

**Trial registration:** ClinicalTrials.gov: Cost-effectiveness of a Transdiagnostic Psychological Treatment for Emotional Disorders in Primary Care (PsicAP). NCT05314920.

## Introduction

Anxiety, depression, and somatoform disorders are three of the most common mental disorders worldwide [1]. According to the World Health Organization [2], prevalence rates for depression and anxiety worldwide are 4.4% (322 million people) and 3.6% (264 million), respectively [3]. These three emotional disorders are major contributors to disability worldwide, with an enormous negative impact on quality of life (QoL) and functional impairment [4–8]. In a study conducted in Spain [9], the estimated 1-year prevalence rates for anxiety disorder, depression, and somatoform disorder in the general population were 6.2%, 4.4%, and 14.7%, respectively; these rates are all significantly above estimated global prevalence rates. Emotional disorders (including depression, anxiety and somatoform disorders) are one of the most common causes of demand for primary care services. Estimated prevalence rates for these disorders in the primary care setting in Spain are 35.8% for depression, 25.6% for anxiety, and 28.8% for somatoform disorders [10]. Given these data, it seems clear that investment in mental healthcare is essential to deal with the increasing demand.

In Spain, consultations with the general practitioner (GP) are usually brief (< 7 minutes on average), and the GP has to decide, in a question of minutes, whether to offer treatment (usually a pharmacological intervention) or to refer the patient to specialized care [11, 12]. Due to the high prevalence rates of mental disorders in the primary care setting, the brief time available for the consultation, and the scant psychological training given to GPs on how to manage these disorders, the end result is commonly a less-than-optimal treatment, mainly prescription of psychotropic medication [11]. Unfortunately, the routine use of these drugs may have several negative effects, including an increased risk of long-term relapse in patients with emotional disorders [13–16], the development of side effects that can negatively influence adherence [14, 17–20], a high treatment dropout rate [21], and even drug dependence [17, 19]. In many cases, this can lead to important personal, social, and economic costs, with a decreased QoL, greater suffering, more frequent use of health services, medication overuse, and an increase in medical leaves from work [22, 23].

According to the Organization for Economic Co-operation and Development (OECD), the use of antidepressants in Spain has increased to 86.2 defined daily doses (DDD) per 1000 habitants [24]. Similarly, the prescription of anxiolytics and hypnotics has also increased over time (57.9 and 33.9 DDD, respectively, in 2019) [24], far exceeding international averages.

A wide range of psychological interventions have proven to be effective for emotional disorders. The National Institute for Health and Care Excellence (NICE) guidelines recommend cognitive-behavioural therapy (CBT) for the management of depression and anxiety [25], as CBT is the psychological approach with the most empirical support for the treatment of emotional disorders [26, 27]. Moreover, studies show that psychotherapy is the preferred treatment among individuals with mental disorders, thus improving adherence and outcomes [28]. Compared to pharmacological treatments, psychotherapy can be less expensive, especially when patients are treated in group format. Moreover, the transdiagnostic approach might further reduce costs by allowing for the simultaneous treatment of patients with different conditions [29]. However, studies have shown that psychological treatments are underused in individuals with emotional disorders [30].

In 2008, the United Kingdom implemented a large-scale program entitled Improving Access to Psychological Therapies (IAPT), which was developed to improve the treatment of emotional disorders for the general population in the primary care setting [31]. The IAPT program offers NICE-recommended low or high-intensity psychological interventions for emotional disorders, with CBT being the most common intervention [32]. In the last decade, the IAPT program has achieved high rates of clinical and functional recovery, with moderate to large effect sizes [33, 34], fewer relapses, and lower long-term economic and social costs than treatment as usual (TAU) [35]. Given the excellent results, that program has been replicated in several other countries [36–38].

Inspired by the IAPT program, several initiatives have been carried out in Spain to test the efficacy of psychological treatment in primary care [39]. A recent randomised controlled trial (RCT)—the PsicAP study—demonstrated the efficacy of adding group transdiagnostic CBT (TD-CBT) to TAU versus TAU alone in primary care patients, obtaining medium effect sizes in the reduction of emotional symptoms [40]. Another RCT conducted in Spanish primary care settings—the PsiBrief study—also achieved medium to high effect sizes in reducing emotional symptoms through a group intervention [41]. Both RCTs were based on a transdiagnostic approach, which focuses on the dysfunctional cognitive-emotional processes shared by various mental disorders. This new approach has proven effective in reducing emotional symptoms and improving QoL [42–45]. It also has the potential to provide an even more significant reduction in costs by using the transdiagnostic group approach to treat individuals with different but related emotional disorders [44].

To our knowledge, however, none of the aforementioned trials has employed an active treatment comparison condition. TAU is commonly used as the comparison condition in clinical trials to assess the effectiveness of a novel intervention. However, once the superiority of a given intervention has been established, it is more appropriate to compare it to active treatment conditions in group formats to control for unspecific factors in the group setting, such as peer support, therapist contact, and expectancy effects. In addition, the main aim of the aforementioned RCTs was to evaluate efficacy, and no cost analyses were conducted, even though such data could be of value in assisting policy decision makers to select the optimal treatment approach based on efficacy and costs.

In the present RCT, we will compare TD-CBT to an active treatment—progressive muscle relaxation (PMR), which has previously proven effective in promoting relaxation states and reducing anxiety and depressive symptoms [46, 47].

### Objectives and hypotheses

The aim of this RCT is to compare the cost-effectiveness of TAU plus group TD-CBT versus TAU plus group PMR. Cost-effectiveness will be determined by the incremental cost-effectiveness ratio (ICER), which is defined as the difference in mean costs between interventions divided by the difference in their effectiveness (through changes in the Patient Health Questionnaire-9 [PHQ-9], Generalized Anxiety Disorder-7 [GAD-7] and Patient Health Questionnaire-15 [PHQ-15] scale scores obtained at completion of the intervention and at the one-year follow-up assessment) in adults with mild to moderate emotional disorders (depressive, anxiety, and somatoform disorders). In both groups, treatment will be delivered in primary care centres.

#### Main hypotheses

We expect that the post-treatment cost-effectiveness ratio will be better in the TD-CBT group than in the PMR group, and that this difference will be maintained at the 12-month follow-up assessment.

#### Secondary hypotheses

Compared to the PMR group, we expect to observe the following outcomes in the TD-CBT group:

1. Better clinical outcomes in terms of anxiety, depression, and somatic symptoms.
2. Greater improvement in social dysfunction and QoL.
3. Post-treatment benefits maintained at the 12-month follow-up assessment.
4. Clinical improvement will be mediated by improvements in cognitive-emotional factors (i.e., rumination processes, worry, attentional and interpretive biases, emotion regulation strategies, and metacognitive beliefs) and therapeutic and group alliance.
5. Individuals with a more severe clinical profile at baseline will benefit more from TD-CBT than those with a mild to moderate clinical profile.

## Methods

The present protocol was registered at ClinicalTrials.gov (NCT05314920). The protocol adheres to the SPIRIT guidelines [48]. For more information see Table 1.

**Table 1:** Items from the World Health Organization trial registration data set.

Data category	Information
Primary registry and trial identifying number	ClinicalTrials.gov NCT05314920
Date of registration in primary registry	April 7, 2022
Source(s) of monetary or material support	Agencia Estatal de Investigación
Primary sponsor	Instituto de Investigación Marqués de Valdecilla
Contact for public queries	CGB [+34-942-202537] [cesar.gonzalezblanch@scsalud.es]
Contact for scientific queries	CGB [+34-942-202537] [cesar.gonzalezblanch@scsalud.es]
Public title	Cost-effectiveness of a Transdiagnostic Psychological Treatment for Emotional Disorders in Primary Care (PsicAP-Costs2)
Scientific title	Cost-effectiveness of Transdiagnostic Group Psychological Treatment for Common Mental Disorders in Primary Care (PsicAP-Costs2): a Randomised Controlled Clinical Trial
Countries of recruitment	Spain
Health condition(s) of problem(s) studied	Anxiety, Depression and Somatoform disorders
Intervention(s)	Experimental: transdiagnostic cognitive-behavioural therapy (TD-CBT) Active comparator: Bernstein and Borkovec progressive muscle relaxation (PMR)
Key inclusion and exclusion criteria	Inclusion criteria: • Patients aged 18 to 65 • Primary care patients seeking treatment for anxiety, depressive or somatic symptoms. • Scores above the predetermined cut-off points on the GAD-7 (> = 10), the PHQ-9 (> = 10) or the PHQ-15 (> = 10 plus a score of 2 in three or more somatic symptoms). • Agreement to participate in the study, with written informed consent. Exclusion criteria: • Major depressive disorder (PHQ-9> 24), severe disability (SDS > 25) and/or any severe mental disorder, including autism spectrum disorders, bipolar disorder, schizophrenia, anorexia nervosa, substance dependence, personality disorder… • Presence of severe or recent suicide attempts • Presence of intellectual disability (IQ < 70). • Current psychological treatment or any type of specialized care related to mental health. • Insufficient Spanish language skills
Study type	Interventional (Clinical Trial)
Date of first enrolment	February, 2020
Target sample size	300
Recruitment status	Recruiting
Primary outcome(s)	• Change in cost-effectiveness data based on the incremental cost-effectiveness ratios (ICER) [Time frame: baseline, immediately after the intervention, and 12-month follow-up]. • Change in cost-utility data based on the incremental cost-utility ratios (ICURs) [Time frame: baseline, immediately after the intervention, and 12-month follow-up] • Effectiveness [Changes in the scores of the various measures used to evaluate symptoms of anxiety, depression and somatizations] ○ Change in depressive symptoms: 9-item Patient Health Questionnaire (PHQ-9) [Time frame: baseline, immediately after the intervention, and 12-month follow-up] ○ Change in anxiety symptoms: 7-item Generalized Anxiety Disorder scale (GAD-7) [Time frame: baseline, immediately after the intervention, and 12-month follow-up] ○ Change in somatic symptoms: 15 item Patient Health Questionnaire (PHQ-15) [Time frame: baseline, immediately after the intervention, and 12-month follow-up]
Key secondary outcomes	• Change in functioning: Sheehan Disability Scale (SDS) [Time frame: baseline, immediately after the intervention, and 12-month follow-up] • Change in treatment satisfaction [Time frame: immediately after the intervention and 12-month follow-up]

### Trial design

**Fig 1:**
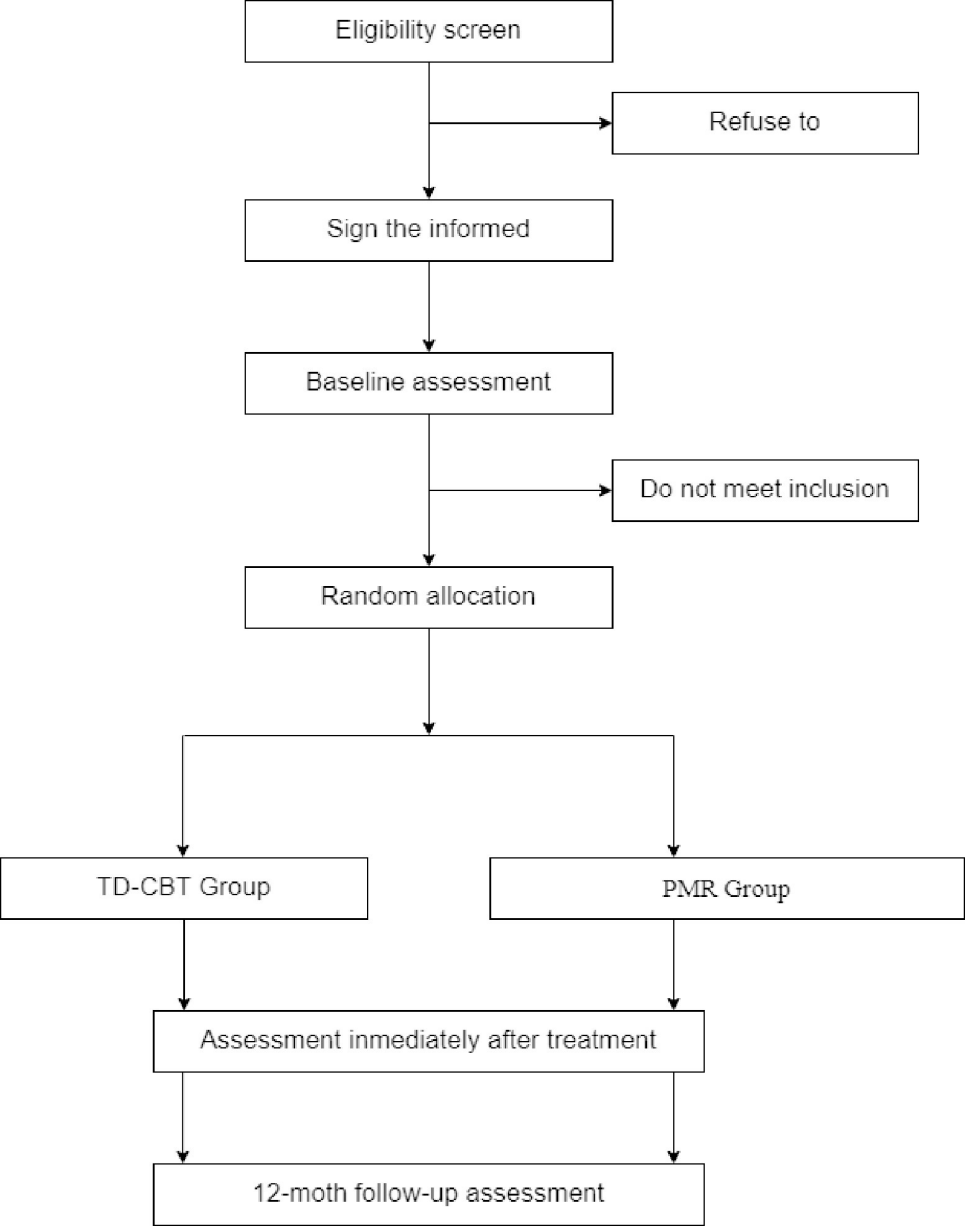
Study design flowchart. TD-CBT: transdiagnostic cognitive-behavioural therapy; PMR: progressive muscle relaxation.

This intervention trial has a randomised, controlled, multicentre, and single-blind design with two parallel groups, a 1:1 allocation ratio, and three assessment time points: pre-intervention, immediately after completion of the intervention, and at 12-months (Fig 1).

### Study settings

This single blind RCT will be conducted in four primary care centres located in Cantabria, Spain, as follows: ‘Camargo Costa’ Health Centre, ‘Camargo Interior’ Health Centre, ‘Sardinero’ Health Centre, and “General Dávila” Health Centre.

### Eligibility criteria

Adult patients (age 18–65 years) with a diagnostic suspicion (based on an unstructured clinical interview with the treating GP) of an emotional disorder (depressive, anxiety, and/or somatoform disorder) will be considered candidates for inclusion. All patients who meet the study criteria will be referred for possible participation.

Prior to enrolment, all candidates will complete a series of screening measures. Patients with scores above the pre-established cut-off points on one or more of the scales (indicating the presence of mild/moderate emotional symptoms) will be invited to participate in the trial. The screening measures include the following instruments: GAD-7 (cut-off score ≥ 10); PHQ-9 (cut-off: ≥ 10); and PHQ-15 (cut-off ≥ 10 plus a score of 2 on three or more somatic symptoms). If, after the candidate has completed all of the screening measures, any doubts remain about eligibility—such as the suspected presence of severe major depression (PHQ-9 ≥ 24) and/or severe disability (Sheehan Disability Scale [SDS] ≥ 25), then a structured clinical interview for DSM IV Axis-I Disorders (SCID-I) will be performed [49]. A specifically-trained clinical psychologist will administer the SCID-I to rule out severe mental disorders (e.g., severe major depression, bipolar disorder, schizophrenia, eating disorders, substance dependence, personality disorders, etc.) and also check for a history of severe and/or recent suicide attempts. If the clinical interview confirms the presence of a severe mental disorder, the patient will be excluded from the study and referred to their GP for appropriate treatment (i.e., referral to a specialized mental health service). Other exclusion criteria include: difficulty understanding the Spanish language, intellectual disability, or current psychological treatment in any setting. The time interval between GP referral to the psychologist and completion of the initial evaluation can range from 1 to 3 months, depending on the time it takes to recruit enough participants to form each treatment group. The first session of the allocated intervention will be scheduled approximately one week after the baseline assessment.

### Interventions

#### Transdiagnostic group Cognitive-Behavioural Therapy (TD-CBT Group)

**Fig 2:**
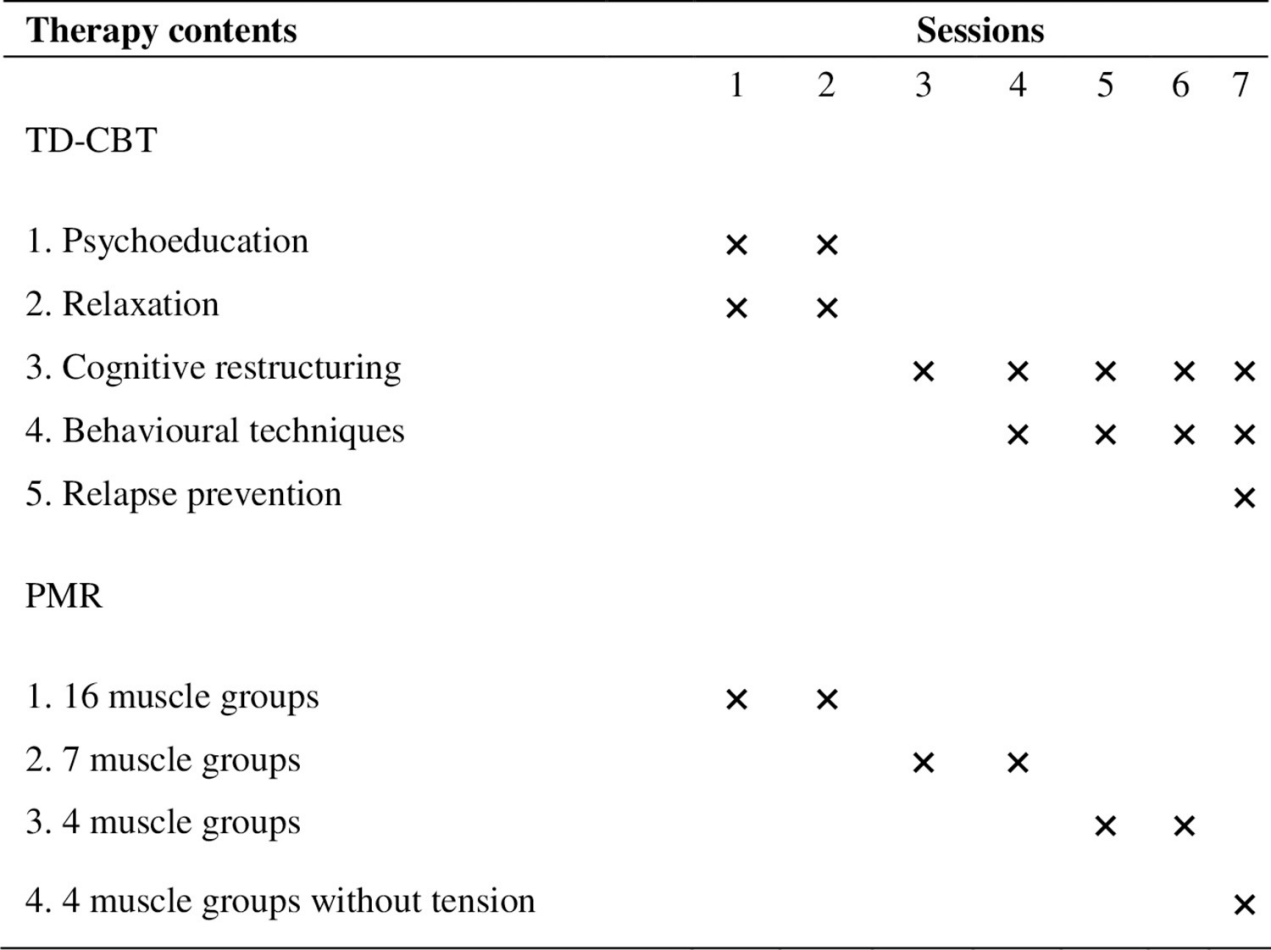
Session timeline for the TD-CBT and PMR groups.

The standardized approach to TD-CBT will be administered by trained clinical psychologists [50]. Both interventions (TD-CBT and PMR) will consist of a total of seven treatment sessions (1.5 hours/session) in groups of approximately 8–12 people. The sessions will be delivered over a 24-week period. Initially, the sessions will be held weekly; over time, the interval between sessions will be gradually increased (see Fig 2 for details). The treatment program in the experimental group (TD-CBT) will include the following content:

#### *Session 1*: *Introduction and Psychoeducation*

In this first session, treatment will begin with psychoeducational training to provide participants with specific, properly-adapted information (oral and written), as well as informational resources (e.g., books, website) on stress, emotions, anxiety, anger, depression, the role of cognitive processes and biases on emotion, emotional learning, emotion regulation, and the relationships between emotion and behaviour, etc. Participants will be encouraged to take an active role in the treatment and to complete the daily homework assignments.

#### *Session 2*: *Psychoeducation and Relaxation*

Relaxation techniques will be introduced, with a mix of Jacobson’s PMR training, abdominal breathing, and imagination. In addition, participants will be asked to practice these exercises daily at home. To help guide this practice, a 30-minute audio recording will be provided. To encourage home practice, the participants will be asked to provide a daily graphic self-registration of the relaxation level experienced throughout the 24 week period.

#### *Sessions 3 and 4*: *Cognitive Restructuring*

These two sessions will focus on cognitive restructuring practice and emotional self-regulation training. Participants will first receive general information about what emotions are and how anxiety, fear, and sadness are manifested (i.e., at the cognitive-subjective, physiological, and behavioural levels). Participants will also learn about the situations in which these responses occur, the reactions that help improve (or hinder) adaptation to the environment, and the appropriate strategies to regulate these reactions. Participants will also learn how easy it is for certain emotional disorders to develop (e.g., due to increased stress levels, or when uncontrolled anxiety reactions are unleashed, attention is focused on the physiological responses it produces).

Participants will be taught, through case presentations, how to detect and change distorted thoughts, irrational beliefs, threats, cognitive biases, emotion regulation strategies, etc., associated with intense emotional states such as anxiety, anger, or other negative states in everyday life. They will learn how they can restore emotional self-regulation by changing information processing (e.g., by encouraging the processing of neutral or positive information, distraction, reducing the importance and magnification of threat or loss, reducing ruminative processes, worry and biases of attention focused on threat or loss, increasing positive self-instructions and perceived self-efficacy, etc.).

#### *Sessions 5 and 6*: *Cognitive Restructuring and Problem Solving*

In these two sessions, the therapy will focus on behavioural techniques to help participants to (re)learn and regain self-control of situations, emotions, and behaviours in their daily lives, which they may have lost the ability to manage properly. This intervention will include a treatment package comprised of several psychological techniques (e.g., exposure) that are started during the group sessions and later practiced daily at home (in addition to relaxation). These techniques include self-observation, stimulus control, reinforced behavioural training, exposure without safety behaviours, coping skills training, among others. Participants will be encouraged to reduce cognitive biases (e.g., attention, interpretation, memory, attribution) that unleash emotional reactions and alter behaviour. To achieve this, homework assignments will focus on rehearsal, practice, reinforcement, correction, progressive exposure, etc.

#### *Session 7*: *Relapse Prevention and Closing*

This session will involve relapse prevention techniques. Participants will learn that relapse does not mean a return to the starting point (i.e., a total loss of therapeutic gains or failure), but rather it represents a new challenge that must be overcome and presents an opportunity to learn more and consolidate previous learning. The participants will be reminded that these challenges/difficulties can be overcome by utilizing the newly learned coping tools, which will have already proven useful to overcome challenges arising during the first seven sessions. Participants will be instructed to use these new skills rather than resorting to the old habits that led to the emotional problems.

### PMR group

PMR is based on the procedure described by Bernstein and Borkovec [51]. PMR consists of learning to sequentially tense and relax different muscle groups throughout the body, directing attention to the feelings associated with tension and relaxation. The same protocol will be maintained across all of the sessions. The session will start with an explanation of the procedure, a brief demonstration of the exercises to avoid possible problems during the relaxation process, a breathing exercise, and the relaxation technique itself. Afterwards, the therapist will spend the final minutes instructing the participants to focus on the feeling of relaxation to verify the effectiveness of the technique. PMR based on the procedure described by Bernstein and Borkovec consists of progressively reducing the number of muscle groups throughout the sessions (16 muscle groups in sessions 1 and 2, seven groups in sessions 3 and 4, and 4 muscle groups in sessions 5 and 6). In session 7, the same four muscle groups will be worked in an effort to achieve a state of relaxation for each muscle group, eliminating the previous tension component (see Fig 2).

The characteristics of the treatment sessions for the PMR group (controls) and the TD-CBT group (intervention) will be the same: seven sessions (1.5 hours each) in groups of approximately 8–12 people delivered over a 24-week period. In each group, the sessions will be led by a clinical psychologist with the same level of experience.

Both groups (TD-CBT and PMR) will also receive TAU by their treating GP. As mentioned above, TAU typically consists of pharmacological treatment and/or unstructured support. The number of consultations during the period of participation in the study will be taken into account.

#### Therapist training

The therapists in this trial will all be psychologists employed by the national health system. The therapists will receive standardized training in the treatment protocols. This training program will consist of a therapist manual, online training sessions, and/or observation of therapeutic sessions supervised by a psychologist specialized in this type of intervention. All therapists will be supervised by a senior clinical psychologist, including follow-up sessions to resolve any doubts or problems that may arise during the intervention.

#### Timeline

**Fig 3:**
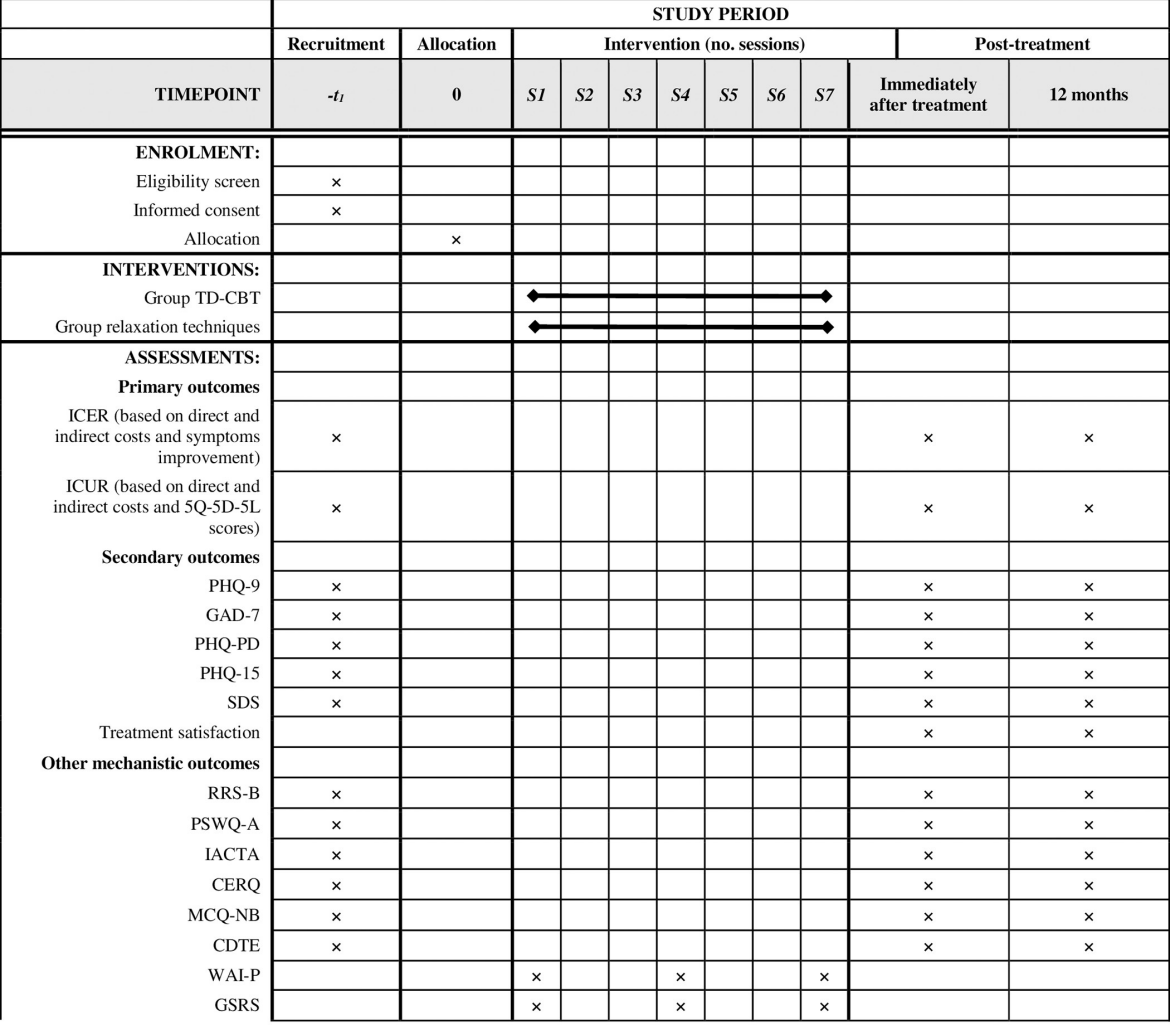
Study timeline according to SPIRIT statement. CDTE: Questionnaire of Cognitive Distortions in Emotional Disorders; CERQ: Cognitive Emotion Regulation Questionnaire; EQ-5D-5L: 5-dimension, 5-level European Quality of Life scale; GAD: Generalized Anxiety Disorder; IACTA-PB: Inventory of Cognitive Activity in Anxiety Disorders-Panic Brief subscale; ICER: incremental cost-effectiveness ratio; ICUR, incremental cost-utility ratio; MCQ-NB: Metacognitions Questionnaire-Negative Beliefs subscale; PHQ: Patient Health Questionnaire; PSWQ-A: Penn State Worry Questionnaire-Abbreviated; RRS-B: Ruminative Responses Scale-Brooding subscale; SDS: Sheehan Disability Scale; TAU: treatment as usual; TD-CBT: transdiagnostic cognitive-behavioural therapy; WAI-P: Working Alliance Inventory Patient Form; GSRS: Group Session Rating Scale.

Fig 3. shows the timing of interventions, as recommended by the SPIRIT guidelines [48].

#### Sample size

The study will recruit a total of 300 participants (150 in each arm) to compensate for expected losses after randomization. Based on previous RCTs conducted in primary care settings, we expect losses to be as high as 30% [52]. Over a 30-month period (February 2020 to June 2023), we will form 10 paired groups (experimental and control) in the four health centres, with a mean of 10 participants in each group. Fifteen GPs will collaborate in the study and each is expected to refer a mean of 10–15 participants for participation, which should provide a sufficient sample size considering that a proportion of these patients will not meet eligibility criteria. We do not foresee any difficulties in recruiting sufficient patients given that each GP conducts an average of 30 patient visits per day (> 7000 visits/year).

#### Recruitment

In Spain, the initial diagnosis of a mental disorder is usually made by the GP in the primary care setting. In other words, this clinical setting is the first level of access. In the first phase (phase 1), GPs will recruit potential candidates whose primary reason for consultation is a suspected emotional disorder (mild or moderate anxiety, depression, and/or somatoform disorders). Next (phase 2), after the candidate has signed the informed consent form, a psychologist trained in using the screening tools to detect anxiety, depressive, and somatoform disorders will administer those instruments in order to identify and select the individuals with mild to moderate symptom intensity (as stipulated in the inclusion and exclusion criteria). Participants who meet the inclusion criteria will be randomly assigned to the experimental or control group by a blinded researcher not involved in evaluation or treatment. Recruitment will be conducted on a rolling basis. Every time 20 to 25 participants have been recruited, they will be randomised to the two groups to ensure that both groups are started at the same time. Candidates who do not meet the inclusion criteria will be excluded and referred back to their treating GP for consultation and treatment. If necessary, patients will be directly referred to the mental health care centre of reference in their area.

In the post-treatment phase, a psychologist not involved in the psychological treatments will repeat the same screening measures as used in the pre-treatment evaluation. This researcher will be blinded to treatment allocation. Neither participants nor the GPs will receive financial compensation for participation in the trial.

#### Allocation

For randomization, the web-based program (GraphPad) will be used. Participants will be assigned to the treatment arms in a 1:1 ratio and will be informed of the allocation by telephone.

#### Blinding

This trial is single-blinded. Evaluators and data managers will be blinded over the entire course of the trial (from the pre-treatment evaluation to the 12-month follow-up). Participants will be asked to avoid disclosing their treatment allocation to the GP and/or the researchers involved in the assessments.

#### Outcomes

All the measures specified below (excluding those related to therapeutic alliance) will be collected at the three pre-specified evaluation times (before treatment, immediately after treatment completion, and at 12 months post-treatment).

*Primary outcome measures*. The primary outcome measures are 1) the ICER, 2) the incremental cost-utility ratio (ICUR) and 3) effectiveness (included in the cost-effectiveness data section).

#### Cost-effectiveness data

Cost-effectiveness will be calculated by the ICER, defined as the difference in mean costs between interventions divided by the difference in their effectiveness, based on changes in the mean scores on the PHQ-9, GAD-7 and PHQ-15 scales obtained immediately after completion of the 7-session intervention and at the 12-month follow-up.

The healthcare data collected will be used for cost calculations. To calculate healthcare-related costs, an *ad hoc* questionnaire will be administered to collect emotional disorder-related healthcare data (public and private healthcare consultations, accidents, medical tests, and sick leaves in the past 3 months). In addition, we will check the patients’ electronic medical records to obtain data on current prescriptions (i.e., drug name and dosage). Likewise, the costs related to the intervention will be calculated by taking into account the cost of consultation with the medical staff in Cantabria, multiplied by the number of hours and sessions of the program and divided by the number of participants in each group. In this way, we will obtain the cost of the complete therapy per person.

#### Cost-utility data

Cost-utility will be measured through the healthcare data collected above and the European Quality of Life Scale (EuroQoL, EQ) [53]. The Quality-Adjusted Life Years (QALY) will be calculated. The utilities (values given by EuroQol) and the ICURs (defined as the difference in mean cost divided by the difference in mean QALYs) will be multiplied by time. The Spanish version of the 5-domain, 5-level EuroQol (EQ-5D-5L) [54, 55] will be used to assess health status in five dimensions (mobility, self-care, daily activities, pain/unease, and anxiety/depression) with five levels of severity (no problems, slight problems, moderate problems, severe problems, and either extreme problems or unable to perform activity). The scores obtained in the different dimensions can be combined to create a 5-digit code describing the patient’s state of health; this system can establish up to 3125 different combinations and therefore different possible health states. This system was converted into a utility score using the value set for Spain from the EQ-5D-5L Crosswalk Index Value downloaded from the EuroQol website (https://euroqol.org/euroqol/).

### Variables to calculate these indices

#### Secondary outcomes measures

Secondary outcomes are the clinical symptoms assessed by the PHQ: depression, anxiety, and somatic symptom severity (PHQ-9, GAD-7, and PHQ-15), QoL, functioning, and treatment satisfaction.

*Depressive symptoms*. The PHQ-9 subscale [56] will be used to assess depressive symptoms. The PHQ-9 is the nine-item depression module of the PHQ [57, 58] that scores the nine DSM-IV depression criteria in the last two weeks. This self-report scale ranges from 0 to 27 (higher scores indicate worse outcomes). The PHQ-9 (cut-off: 10 points) has a sensitivity and specificity of 95% and 67%, respectively, for depression [59].

*Anxiety symptoms*. The GAD-7 scale [60] will be used to assess common anxiety. Scores on this self-report scale range from 0 to 21 points, with higher scores indicating a greater presence of anxiety symptoms. With a cut-off of 10 points, the sensitivity and specificity of the GAD-7 is 87% and 78%, respectively [61].

*Somatic symptoms*. The PHQ-15 [62] will be used to assess somatic symptoms. This scale is the somatoform module of the PHQ and scores symptoms present in the past four weeks. The scale is composed of 15 self-report items, ranging from 0 to 30, with higher scores indicating worse outcomes. For this study, we will use a cut-off point of 10 plus a score of 2 on three or more somatic symptoms. This tool has been validated in psychiatric outpatients in Spain (α = .78) [63].

*Functioning*. The SDS [64] will be used to assess functioning. The SDS is a five item self-report scale composed of three main domains (work, family, and social functioning) and two additional items (perceived stress and perceived social support). Scores range from 0 to 50 points, with higher scores indicating a worse outcome. This tool has been validated in the Spanish population [65] and showed good psychometric properties in primary care patients (α = .83) [66].

*Treatment satisfaction*. An *ad hoc* questionnaire will be used to assess treatment satisfaction at two time points: 1) immediately after treatment completion (post-treatment) and at 12-months of follow-up.

#### Other mechanistic outcomes

Measures of cognitive-emotional processes (i.e., worry, rumination, metacognition, emotion regulation, attentional and interpretative biases, and cognitive distortions), and therapeutic alliance will be considered as mechanistic outcomes.

*Rumination*. The Ruminative Responses Scales (brooding subscale) (RRS-B) [67] will be used to assess rumination. The RRS-B is composed of five self-reported items, with total scores ranging from 5 to 20 points. Higher scores indicate worse outcomes. The RRS-B has been validated in the Spanish population [68], with an internal consistency of α = .79 [69].

*Worry*. The Penn State Worry Questionnaire–Abbreviated (PSWQ-A) [70] will be used to assess pathological worry as an uncontrollable and general state. The scale is composed of eight self-reported items, with total scores ranging from 5 to 40 points. Higher scores indicate worse outcomes. This tool has been validated in a Spanish population [69], showing good psychometric properties in primary care (α = .90) [71].

*Attentional and interpretative biases*. The Inventory of Cognitive Activity in Anxiety Disorders–Panic Brief version (IACTA-PB) [72] will be used to assess attentional and interpretative biases according to Eysenck’s four-factor theory [73]. The scale is composed of five self-reported items, with total scores ranging from 0 to 20 points. Higher scores indicate worse outcomes. The IACTA-PB has shown good psychometric properties (α = .87) [69].

*Emotion regulation strategies*. The 27-item Cognitive Emotion Regulation Questionnaire (CERQ-27) [74] will be used to assess the specific cognitive emotion regulation strategies that a person uses to face stressful events. Scores on each item range from 1 ("almost never") to 5 ("almost always"), indicating how often the participant thinks as described in the item. The CERQ-27 has been validated in the Spanish population [75] and has shown good psychometric properties (internal consistencies range from α = .72 to α = .88) [74].

*Metacognitive beliefs*. The Metacognitions Questionnaire (negative beliefs subscale) (MCQ-NB) [76] is a short form of the original MCQ [77]. It will be used to assess beliefs about the individual’s own thought processes. The scale is composed of six self-reported items, with total scores ranging from 6 to 24 points. Higher scores indicate worse outcomes. The MCQ-NB has been validated in the Spanish population [78], showing good psychometric properties (α = .82) [69].

*Cognitive distortions in emotional disorders*. The Cognitive Distortions in Emotional Disorders Questionnaire (CDTE) [79] will be used to assess the frequency of certain cognitive biases. This scale includes 16 self-reported items that measure the presence of four factors: sustained attention bias, divided attention bias, magnification interpretational bias, and catastrophization interpretational bias. Scores on each item range from 0 to 4 points. This tool has shown good psychometric properties in all four factors (α ≥ .94) [79].

*Alliance*. The Working Alliance Inventory Patient Form (WAI-P) [80] is a 36-item self-report scale that will be used to assess perceived therapeutic alliance. Total scores range from 36 to 252 points, with higher scores indicating better alliance between the patient and clinician. This tool has been validated in the Spanish population, showing good psychometric properties (α = .96) [80]. The Group Session Rating Scale (GSRS) [81] is a four-item self-reported scale that assesses alliance with the group. Total scores range from 0 to 40 points, with higher scores indicating better alliance between the patient and the therapy group.

Data management. Scores obtained from both physical and online format questionnaires will be tabulated, exported, and included in the IBM-SPSS Statistical software program, v. 23 (IBM Corp., Armonk, N.Y., USA).

#### Statistical methods

*Analysis of clinical effectiveness*. Data on treatment effectiveness will be analysed on an intention-to-treat (ITT) basis through changes in the scores on the PHQ-9, GAD-7 and PHQ-15 scales obtained at completion of the 7-session intervention versus scores obtained at the 12-month follow-up. Thus, all participants who have been enrolled in the study will be considered in the analysis, even if they failed to comply with the protocol. This approach will allow us to maintain randomization until the end of the study, thus decreasing the probability of biased results. Mixed-model repeated measures (MMRM) analyses will be used to compare change in primary, secondary, and other mechanistic outcomes measures between the two treatment groups over the 12-month follow-up. MMRM uses all available data (including participants with partial data) to estimate treatment effects. Time (baseline, post-treatment, and at 12 months) will be the within-person predictor and treatment group (TD-CBT+TAU vs. PMR+TAU) will be used as the between-person predictor. The effect sizes (Cohen’s *d or Rosenthal’s r*, as appropriate) in both groups on the various dependent variables will be calculated, as well as their accuracy (95% confidence intervals [CI]), taking into account the number of sessions received. Recovery, reliable recovery, and deterioration rates will also be calculated. Recovery rates will be determined by identifying the percentage of patients with scores below the cut-off point on any of the three scales (GAD-7, PHQ-9, and PHQ-15) at the post-treatment or 12-month assessment. The reliable recovery index will be calculated using a change score based on the standard deviation and Cronbach’s alpha for each measure to consider scale measurement errors [82]. Secondary analyses will include moderation and mediation analyses using the Hayes’s Process Macro for SPSS. When necessary, missing data will be treated through appropriate methods (e.g., multiple imputation).

*Description of the cost analysis*. The economic evaluation will be carried out through self-report questionnaires and electronic clinical records before the start of treatment, at treatment completion, and at the 12-month follow-up. Direct costs will be calculated by adding the costs arising from the use of psychopharmacological medicines (anxiolytics, hypnotics, sedatives and/or antidepressants), the number of visits, and health-related services and personal costs, and medical examinations carried out. The cost of medicines will be estimated by determining the price per milligram (mg) according to Vademecum International and multiplying the price per mg (€/mg) per daily dose, and the number of days of treatment with medicines. Costs related to the use of public health services and health tests will be calculated based on the rates published in the official gazette (*Boletín Oficial de Cantabria)* of the government of Cantabria (O. SAN/35/2017). Indirect costs will be estimated by the number of days on sick leave from work multiplied by the minimum daily salary in Spain at that time. If a replacement worker is required, the cost will also be included by multiplying the number of days that the worker has been replaced by the daily minimum salary at that time. Total costs will therefore be the sum of direct and indirect costs. Uncertainty surrounding the incremental costs and effects will be estimated by non-parametric bootstrapping with 5000 replications. The 95% CI around the mean cost differences will be estimated using the approximate bootstrap confidence algorithm followed by Student’s t test. Bootstrapped incremental cost-effect pairs will be plotted on a cost-effectiveness plane showing differences between the two interventions in effects (horizontal axis) and costs (vertical axis).

*Cost-effectiveness analysis*. The cost-effectiveness analysis will be carried out by calculating the ICER, defined as the difference in the average cost divided by the increase in effectiveness between the different therapeutic alternatives. Specifically, effectiveness will be calculated based on changes in the scores on the questionnaires that evaluate symptoms of anxiety, depression and somatizations (PHQ-9, GAD-7 and PHQ-15). Cost-effectiveness acceptability curves (CEACs) will be estimated to examine the probability that the intervention is cost-effective compared to PMR+TAU for a range of willingness-to-pay thresholds. The analysis will involve adopting multilevel modelling statistical techniques, taking into consideration clustering in the cost‐and‐effect data. We will apply the threshold suggested by the National Institute for Health in Spain, which is €20,000–25,000/QALY [83].

*Cost-utility analysis*. The cost-utility analysis refer to the use of the intervention health-related services, which are collected through subjective patient information and medical history. This allows the participants to express preferences based on the value they place on their health status, thus depending on a societal perspective. The analysis will be carried out using the EuroQoL, EQ-5D-5L [54, 55]. We will calculate the QALYs and ICURs. Given the relatively short follow-up period (12 months post-intervention), neither costs nor results will be subject to discount.

*Sensitivity analysis*. A sensitivity analysis will be carried out to test the robustness of cost-effectiveness and cost-utility results with the purpose of exploring whether plausible changes in the costs of the key variables (i.e., medication and therapists) affect the results of the analysis. More specifically, we will evaluate two possible scenarios: a 20% reduction in the cost of drugs and a 20% increase in the costs of group psychological sessions.

#### Monitoring

The trial will not have a data monitoring committee, since the potential harms are limited to medications routinely prescribed by GPs in the primary care setting. The progress of the trial will be monitored through regular contact and meetings among the treating psychologists and the principal investigator. All updates will be published through online registries: ClinicalTrials.gov (NCT05314920).

### Ethics and dissemination

#### Ethics approval

The project has been approved by the Clinical Research Ethics Committee with Medical Products of Cantabria (ref. 2020.011). All professionals involved in this study have agreed in writing to adhere to the Helsinki Declaration and to Spanish law. All participants will be asked to provide verbal and written informed consent for study participation.

#### Consent and confidentiality

A complete description of the study will be provided to participants. The written informed consent will provide details about the study and the risks involved. Data confidentiality will be guaranteed by using numeric codes. All data will be treated in accordance with Spanish data security laws. Data will be stored in a protected central server that will be only accessible for direct members of the study team.

#### Dissemination policy

The main findings of this trial will be disseminated through scientific articles, academic conferences, and science communication activities.

## Discussion

The proposed RCT seeks to expand on the preliminary results obtained by the PsicAP [40] and PsiBrief projects [41]. In this new RCT, we aim to repeat the strengths of those previous RCTs while adding new components: the comparison of TD-CBT with a new active treatment modality (PMR), and by adding a cost-effectiveness analysis of the two interventions (TD-CBT+TAU vs. PMR+TAU). The proposed study protocol also overcomes some of the limitations of those previous studies, mainly the absence of a rigorous cost-effectiveness analysis. Although efficacy is a vital measure, it is clear that the interventions should also be evaluated and compared in terms of costs. The PsicAP-Costs2 RCT will allow us to compare the effectiveness and cost-effectiveness of two active psychological treatment modalities. The use of an active comparator (PMR group) with similar format characteristics to the experimental condition (TD-CBT group) will enable us to control for nonspecific effects of the group therapy.

Based on the results of the PsicAP trial [40], we expect that adding group TD-CBT to TAU in primary care will be more cost-effective than TAU plus PMR, and we also expect the gains to be maintained during the 12-month follow-up. If this trial is successful, the widespread utilization of an efficacious, cost-effective treatment would help improve access to psychological treatment for emotional disorders in primary care centres within the national health system. This would also significantly reduce the cost of treating emotional disorders in this setting. In addition, positive trial results might help GPs to better understand how effective psychological treatments can be in preventing relapses and in treating emotional symptoms. In turn, this would reduce the number of primary care patients requiring treatment, and would also improve the well-being of the population. It will also provide evidence to assist health policy decision makers to analyse the economic processes in this field.

The inclusion of an active comparator will enable us to conduct secondary moderation analyses to determine which sociodemographic and clinical subgroups most benefit from adding TD-CBT to TAU or, alternatively, the groups that obtain equivalent (or greater benefits) from a simpler treatment such as PMR. Secondary mediation analyses will provide valuable data on the role of putative mediators such as cognitive-emotional and alliance related factors.

### Anticipated limitations

We expect to find the following limitations. First, it seems likely that, due to the high workload in primary care, together with the limited time that GPs have between consultations, patient recruitment may be challenging. However, to reduce this risk, four different health centres will be involved in this trial, thus expanding the potential population for recruitment. Second, due to the ongoing COVID-19 pandemic, it may be difficult to offer treatment in a group format. To reduce this risk, the group sessions will be conducted in large, well-ventilated spaces. In addition, the number of participants per group could be reduced if necessary to respond to possible changes in the health safety rules. Third, there is a risk of high dropout rates and missing data, especially at the 12-month follow-up. To compensate for this risk, the maximum expected attrition rate has been estimated at 30% (leaving a large margin of error) based on data from similar RCTs conducted in primary care settings; in addition, ITT and MMRM analyses will be used. Finally, the use of self-report screening tools (rather than semi-structured interviews conducted by clinicians) to determine symptom severity is a limitation. However, all the scales used in this project have been validated in Spanish primary care and are considered reliable instruments.

### Future directions

This trial aims to compare the cost-effectiveness of adding an empirically-supported, active, transdiagnostic treatment (TD-CBT) in primary care for patients with mild to moderate emotional disorders. The project has the potential to transform clinical practice and policy for people with emotional disorders. If the trial is successful, it would imply that clinical psychologists should become more actively involved in primary care to deliver psychological treatments such as TD-CBT. This would lead to shorter waiting lists for individuals with emotional disorders, which in turn would reduce the time that these patients are forced to live with disability. This would also significantly improve patient wellbeing, and would optimize resources in primary care and improve mental health services.

In the future, we hope to adapt TD-CBT for the treatment of children and adolescents. This is important given that the evidence shows that emotional disorders in the adult population often begin during childhood and adolescence. Moreover, these treatments can also be adapted for older people (≥65 years) to improve QoL and reduce health and social costs.

Once the trial has been completed, secondary analyses of the study data will allow us to identify moderators and mediators of treatment outcomes, which might help to personalize treatment and gain a better understanding of the processes underlying response to the therapy [27]. In short, this therapeutic approach offers the potential for a significant reduction in costs and waiting lists, together with long-lasting improvements in the QoL and well-being of these patients.

## Supporting information

S1 Checklist SPIRIT 2013 checklist: Recommended items to address in a clinical trial protocol and related documents*. (DOC)

S1 Appendix Model consent form. (DOCX)

S1 File (DOCX)

S2 File (DOCX)

## List of abbreviations

CBT: Cognitive Behavioural-Therapy
CDTE: Questionnaire of Cognitive Distortions in Emotional Disorders
CERQ: Cognitive Emotion Regulation Questionnaire
DDD: Defined Daily Doses (per 1000 habitants)
DSM: Diagnostic and Statistical Manual of Mental Disorders
EQ-5D-5L: 5-domain, 5-level European Quality of Life Scale
GAD: Generalized Anxiety Disorder
GBD: Global Burden of Disease
GP: General Practitioner
GSRS: Group Session Rating Scale
IACTA-PB: Inventory of Cognitive Activity in Anxiety Disorders-Panic Brief version
IAPT: Improving Access to Psychological Therapies
ICER: Incremental Cost-Effectiveness Ratios
ICUR: incremental Cost-Utility Ratio
IDIVAL: Valdecilla Biomedical Research Institute
ITT: Intention-to-Treat
MCQ-NB: Metacognitions Questionnaire-Negative Beliefs subscale
NICE: National Institute for Health and Care Excellence
OECD: Organization for Economic Co-operation and Development
PD: Panic Disorder
PHQ: Patient Health Questionnaire
PMR: Progressive Muscle Relaxation
PsicAP: *Psicología en Atención Primaria* (Psychology in Primary Care)
PSWQ-A: Penn State Worry Questionnaire-Abbreviated
QALYs: Quality-Adjusted Life Years
RCT: Randomised Controlled Trial
RRS-B: Ruminative Responses Scale-Brooding subscale
SDS: Sheehan Disability Scale
TAU: Treatment As Usual
TD-CBT: Transdiagnostic Group Cognitive Behavioural Therapy
UI: Uncertainty Interval
WAI-P: Working Alliance Inventory Patient Form
WHOQOL: World Health Organization Quality of Life Instrument
