# Supplementary material for: Cost-effectiveness of transdiagnostic group cognitive behavioural therapy versus group relaxation therapy for emotional disorders in primary care (PsicAP-Costs2): Protocol for a multicentre randomised controlled trial
Source: PLoS One. 2023 Mar 16;18(3):e0283104. doi: 10.1371/journal.pone.0283104 (PMC10019745; doi:10.1371/journal.pone.0283104)
Supplement: S1 Appendix — (DOCX) [file pone.0283104.s002.docx]

**Appendix**

**Appendix 1. Model consent form.**

INFORMED CONSENT

**CLINICAL TRIAL TITLE**: Efficacy of transdiagnostic group psychological treatment versus relaxation group treatment for common mental disorders in primary care: a low-intervention randomised controlled clinical trial.

I (name and surname)

..........................................................................................................................

I have read the information sheet provided to me.

I was able to ask questions about the study.

I have received enough information about the study.

I have spoken to: ..................................................................... (name of investigator)

I understand that my participation is voluntary.

I understand that I can withdraw from the study:

1º Whenever I want

2º Without having to give explanations.

3º Without any repercussions on my health care.

- I freely give my agreement to participate in the study and consent to the access and use of my data under the conditions detailed in the information sheet.

YES □ NO □

**Patient's signature: Investigator's signature:**

**Name: Name:**

**Date: Date:**
