# Supplementary material for: Cost-effectiveness of transdiagnostic group cognitive behavioural therapy versus group relaxation therapy for emotional disorders in primary care (PsicAP-Costs2): Protocol for a multicentre randomised controlled trial
Source: PLoS One. 2023 Mar 16;18(3):e0283104. doi: 10.1371/journal.pone.0283104 (PMC10019745; doi:10.1371/journal.pone.0283104)
Supplement: S1 File — (DOCX) [file pone.0283104.s003.docx]

**OFFICIAL TITLE:** COST-EFFECTIVENESS OF TRANSDIAGNOSTIC GROUP PSYCHOLOGICAL TREATMENT VERSUS RELAXATION GROUP TREATMENT FOR COMMON MENTAL DISORDERS IN PRIMARY CARE (PSICAP-COSTS2): RANDOMIZED CONTROLLED CLINICAL TRIAL

**BRIEF TITLE:** COST-EFFECTIVENESS OF TRANSDIAGNOSTIC PSYCHOLOGICAL TREATMENT FOR EMOTIONAL DISORDERS IN PRIMARY CARE (PSICAP-COSTS2)

**Investigators**

- César González-Blanch Bosch, PhD.

Mental Health Centre, Marqués de Valdecilla University Hospital - IDIVAL, Santander, Spain.

Faculty of Health Sciences, Universidad Europea del Atlántico, Santander, Spain

- Antonio Cano Vindel, PhD.

Faculty of Psychology. Complutense University of Madrid, Madrid, Spain

**Contacts**

Contact: César González-Blanch, PhD

Tel: +34-942-202537 Fax: +34-942-203447

E-mail: [cesar.gonzalezblanch@scsalud.es](mailto:cesar.gonzalezblanch@scsalud.es)

**Abstract:**

The aim of this study is to test the cost-effectiveness and cost-utility of adding a transdiagnostic group cognitive-behavioural therapy (TD-CBT) to treatment as usual (TAU) for emotional disorders in primary care . A single-blind randomized controlled clinical trial will be conducted to compare the TD-CBT group therapy plus TAU to progressive muscle relaxation (PMR) group plus TAU in individuals, aged 18 to 65, with emotional disorders in four primary care centres located in Cantabria, Spain. The study will take a societal perspective. Psychological assessments will be carried out at baseline, post-treatment, and 12-months follow-up. The assessments will include measures of clinical symptoms (anxiety, depression, and/or somatic), dysfunction, cognitive-emotional factors (ruminative processes, pathological concern, attentional and interpretative biases, emotion regulation strategies and meta-cognitive beliefs), and satisfaction with the treatment received. Data on health service use, including medication and days of absence from work, will be collected from electronic medical records. The primary outcomes are the incremental cost-effectiveness ratios (ICER) based on the difference in mean costs and effectiveness between interventions and incremental cost-utility ratios (ICURs) based on health-related quality of life at post-treatment and 12-month follow-up. Secondary outcome measures include clinical symptoms, quality of life, functioning and treatment satisfaction. Bootstrap sampling will be used to assess the uncertainty of the results. Secondary moderation and mediation analyses will also be conducted. In addition, in sessions' number 1, 4 and 7 of both treatment arms, two questionnaires will be administered that collect therapeutic alliance and group satisfaction. The main study hypothesis is that adding TD-CBT to TAU in primary care will be more cost-effective than TAU plus PMR. In addition, these gains will be maintained in the 12-month follow-up. If it is successful, the dissemination of cost-effective treatment can help to overcome problems in accessing psychological treatment for emotional disorders in the context of an increasing demand for mental healthcare in primary care.

**Pathologies:**

Emotional disorders (depression, anxiety and somatoform disorders)

**Key words:**

transdiagnostic therapy

progressive muscle relaxation

emotional disorders

primary care

cost-effectiveness analysis

cost-utility analysis

brief psychological treatments

cognitive behavioural therapy

randomized controlled trial

depression

anxiety

somatoform disorders

**BACKGROUND AND CURRENT STATUS OF THE TOPIC AND BIBLIOGRAPHY**

**The extent of mental disorders**

Epidemiological studies in Spain indicate that around 1 in 5 people will present a mental disorder during their lifetime [1] and 8.4% during the last 12 months. Anxiety disorders (6.2%) and mood disorders (4.4%) are the most frequent in the last 12 months [1]. In Cantabria, the point prevalence in some epidemiological studies is 14.7%. [2] In the case of Primary Care (PC) health centres, it can be estimated, based on the different epidemiological studies in our environment, that around 30-35% of the people they attend have a mental disorder. At this level of care, anxiety disorders are the most frequent, followed by depressive disorders. [3]

**The cost of common mental disorders**

In developed countries, 38% of illnesses are mental disorders [4], particularly affecting people of working age where they account for 50% of all illnesses. Recent studies in Europe rank mental disorders as the leading cause of loss of quality of life expressed as Disability Adjusted Life Years (DALYs) and as the main reason for disability pension claims. [5] In Spain, mental disorders are the second most frequent cause (after diagnoses of osteo-muscular origin) of temporary incapacity (TI) processes managed by PC physicians. Moreover, according to data from Cantabria, more than 80% of the diagnoses included in mental health TI reports correspond to "neurotic" or "minor" disorders. [6] Paradoxically, the mental disorders with the best prognosis (i.e. those related to anxiety, depression and adaptive disorders) are one of the main sources of expenditure for TI benefits, as they combine two characteristics: high frequency and very long duration. [7]

The economic costs of mental disorders were estimated for 2010 in the European Union at €461 billion, excluding the costs of dementia and neurological disorders; the main part of this figure corresponds to indirect costs linked to lost productivity and disability pensions. [8] In Spain, the social cost of mental disorders (excluding dementia and other neurological disorders) accounted for 46 billion euros in 2010, with anxiety-related disorders and depression, at more than 10 billion each, accounting for the largest shares of this expenditure. [9]

These costs are underestimated because they do not take into account, among other things, the effect of comorbidity of mental disorders on people with physical illnesses. For example, people with chronic physical illnesses (asthma, cancer, COPD, arthritis, hypertension, diabetes, heart attacks and heart problems) with comorbid anxious-depressive disorders have an average monthly medical expenditure between 33-169% higher than the average for these illnesses, excluding out-of-pocket costs for mental health services. [10] Moreover, mental disorders increase the risk of developing some physical illnesses. [11] People with depression have an increased risk of developing a heart problem; after a heart attack, each additional depressive symptom that develops increases the risk of another heart attack by 15%. [12]

Excess mortality is well established among patients with mental disorders compared to the general population. [13,14,15] In particular, the risk of suicide is increased for all mental disorders, not only for the most severe disorders, but the adaptive disorders, a residual, quintessentially mild diagnostic category, have a suicide risk more than 10 times that of the general population. [16] Psychological autopsies indicate that around 90% of suicides have a mental disorder as the main motive. [17] In Spain, suicide is the most likely cause of death for a man aged 20-49. Taking the figures as a whole, the number of deaths from suicide per year, 3,870 people in 2013, are higher than those from traffic accidents, falls, murder or drugs. [18] Finally, it has been noted that contact with PC professionals in the time prior to suicide is common; however, although three out of four suicide victims had contact with PC professionals within a year of suicide, only a third of victims had had contact with mental health services. [19]

**Mental disorders are undertreated, late and mistreated**

A review of epidemiological data drawn from 27 studies involving 150,000 people from 16 European countries highlighted, in addition to the large size and range of psychosocial disturbances associated with mental disorders, that mental disorders have a relatively low rate of treatment. Only 26% of all cases with mental disorders had had formal health care consultations for mental health reasons in the last month. [20] Among treated cases with mental disorders, there was a long delay between onset of the disorder and first contact for treatment and only a small number of patients received minimally adequate intervention. [21]

**The efficacy of cognitive behavioural therapy**

Several meta-analyses have shown that CBT is effective for common mental disorders, such as anxiety and depression. Large effect sizes have been found for disorders such as unipolar depression, generalised anxiety, panic disorders with or without agoraphobia, social phobia or post-traumatic stress disorder. [22] In the case of depression, CBT, the most widely researched form of psychotherapy, a recent meta-analysis found a moderate-large effect size (Hedges g = 0.71), which corresponds to a number needed to treat (NNT) of 2.6.

There is strong evidence that the efficacy of psychological therapy and CBT in particular is equal or greater than pharmacological treatments for the most common affective and anxiety disorders, as well as having greater long-term effectiveness. [22,24-25] In particular, the benefits of psychological approaches include a reduction in symptoms associated with anxiety and depression, a decrease in the risk of relapse, stability of long-term treatment effect, and high rates of recovery, prevention of chronicity and decreased health costs, in terms of medical consultations and medication use, as well as sick leave. [26]

In the case of research on the effectiveness of CBT in the PC setting, although the number of studies is smaller, a recent meta-analysis showed that CBT-based treatment for depressive and anxiety disorders is more effective than no treatment (d =0.59) and treatment as usual in PC (d = 0.48). [27] Of note, there is substantial evidence that psychological treatments using the CBT approach in depressed patients in PC treated with less resource-intensive interventions achieve similar outcomes to those of more intensive treatments. [28] This is especially relevant in view of the fact that CBT-based group treatment may be a particularly cost-effective modality. A meta-analysis of 32 studies of adults with depression found that after the intervention depression levels were significantly reduced with a moderate effect size (g=0.40) compared to controls, and these results were maintained at 6-month follow-up. [29] The typical format in the included studies was groups of 6-10 participants, with 1-hour therapy extending over 2-8 weeks. Similarly, research in 14 studies with psychological group therapy in PC (n = 1,217), comparing treatment as usual in PC with psychological group therapy plus treatment as usual, has shown clinical improvement with a considerable effect at the end of treatment (d = 0.55). [30] Research in this field in Spain is scarce. Some studies point to the efficacy of group psychological interventions based on CBT in PC for depression in both the short and long term [31], and also in the case of anxiety disorders. [32] Although the methodological weaknesses of these studies make it necessary to be cautious with the interpretation of the results.

Based on the results of extensive research on the effects of psychological treatments, the *National Institute for Health and Care Excellence* (NICE) guidelines recommend the use of psychological treatments, particularly CBT, in PC before pharmacological treatments for mild and moderate major depression, generalised anxiety disorder, post-traumatic stress disorder, obsessive-compulsive disorder and panic disorder (i.e. for the most common mental disorders). [33]

Clinical guidelines emphasise the conjunction of empirical evidence with patient values and preferences in the selection and application of treatments. Patients with mental disorders as a whole show a 3-fold greater preference for psychological than pharmacological treatment. [34] A recent meta-analysis states that pharmacological treatment is 1.76 times more likely to be refused and 1.20 times more likely to be abandoned than psychotherapy [35] In this regard, it is relevant to note that patient preferences can directly affect therapy outcomes; there is growing evidence that providing patients with their preferred treatment is associated with greater adherence and better clinical outcomes. [36] Thus, it has been observed that when PC patients with depression received their preferred treatment, their recovery as measured by the HAMD-17 depression scale increased significantly: +2.9 points for pharmacological treatment and, in the case of group CBT, by +8.0 points, which should be considered clinically significant. [37]

**A novel approach to the treatment of mental disorders - the transdiagnostic model**

Although research has demonstrated the effectiveness of psychological treatments for common mental disorders, only a small proportion of those suffering from these mental disorders receive them. Several reasons have been put forward for the underuse of psychological treatments, such as stigma, lack of training of professionals in psychological treatments or, among other reasons, the multiplicity of treatment protocols for different disorders, which often have significant comorbidity, making it difficult for the clinician to decide which protocol to follow. The high rates of psychiatric comorbidity, which in some disorders are above 80% [38], the difficulty in delimiting specific aetiologies for the more than 400 mental disorders listed in diagnostic manuals, and the questionable validity of these manuals have given rise to new proposals for classification and intervention based on transdiagnostic models [39]. This perspective assumes that different emotional disorders share important characteristics and proposes that this overlap emerges from common biological and psychological vulnerabilities that, associated with psychosocial stressors, may result in different manifestations of the same vulnerability. [40] The transdiagnostic perspective emphasises the role of emotion regulation as a key concept for understanding and treating emotional disorders. The concept of emotion regulation refers to the strategies that people engage in to modify the emotional experiences they undergo. Inappropriate emotion regulation strategies such as rumination and avoidance have been found to play an important role in the onset and maintenance of anxiety and depressive symptoms prevalent in common mental disorders. [41] The transdiagnostic model draws on the contributions of cognitive-behavioural theories to incorporate common principles of empirically supported treatments, namely, re-evaluation of negative interpretations, modification of maladaptive behaviours, prevention of emotional avoidance, and use of emotional exposure procedures for feared situations and re-activation of behaviour in low mood. [42] These mechanisms have been shown not only to change behaviour and emotional experience, but also to produce changes in brain function. [43]

**Programmes to facilitate access to psychological treatments**

Based on the above evidence, facilitating access to psychological treatments has become a priority. It is worth highlighting the initiative launched by the UK government in 2007 to offer CBT-based treatments for the most common mental disorders in the PC setting (mainly anxiety and depression), in accordance with the recommendations of NICE guidelines. [44] The programme was called Improving Access to Psychological Therapies (IAPT) and has been the largest effort to date worldwide to disseminate evidence-based psychological treatments to the general population through PC services. Previously, two pilot projects were run for 13 months with clinical outcomes that largely met expectations. [45,46] A large number of patients with these disorders were treated with low-intensity interventions (including different group treatment modalities), which is particularly useful for achieving high cost-effectiveness. At initial assessment, 55-56% of patients who had attended at least two sessions, including the assessment interview, were classified as recovered when they left services, and treatment gains were largely sustained at 10-month follow-up. At further evaluation, effect sizes of d=1.39 for anxiety problems and d=1.41 for depression problems were achieved. In addition, high recovery rates (76% for depression and 74% for anxiety), decreased risk of relapse and maintenance of positive long-term outcomes were achieved. [46] Of the patients who completed the course of treatment in IAPT, 45% recovered (based on a strict dual-criterion, score below the clinical threshold for anxiety and depression), and an additional 16% showed improvement just below full recovery. [47]

**The situation in Spain with regard to the treatment of mental disorders**

A similar initiative to disseminate evidence-based psychological treatment has not been developed in Spain, despite the fact that 65% of people diagnosed with a mental disorder in the last twelve months have not received any health treatment in the last year [48] and the burden of mental illness is greater than that of physical illness and its onset precedes physical illness. [49] Although almost 60% of people with mental disorders visit their GP, psychological treatment is not usually provided in this setting, so this type of treatment only reaches a small percentage of patients, who are referred to Specialised Care Services (Mental Health).

The proportion of cases diagnosed with an anxiety or mood disorder in the last twelve months that receive minimally adequate treatment according to scientific evidence is low, being only 31.8% in specialised care (10.8% in the case of psychological treatment) and 30.5% in PC [50] (the lowest percentages among the countries in our environment). Similarly, psychological treatment is applied to only 0.9% of cases with an anxiety disorder in the last twelve months [48], despite being the treatment of choice [34]; 27% of these patients receive psychological and pharmacological treatment, while 33% receive exclusively pharmacological treatment and 39% of the minority with mental disorders who come to consult health services do not receive any treatment for this type of disorder. [48]

Different factors may explain this situation, such as the training of professionals, the time required, the overload of care, the attitudes of professionals or the organisation, or the relationship between PC and specialised care. What is certain is that empirically supported treatments involve sessions of at least 1 hour and with a weekly or fortnightly frequency, at least initially. These characteristics exceed the resources that GP have to care for their patients. This explains the tendency to use pharmacological treatment as almost the only therapeutic option, even in cases where it is not the treatment of choice, it is not the patient's preference among the available treatments or the patient has not responded favourably to it in a first attempt. In this line, the Mental Health Strategy of the National Health System recognises that the marked tendency to over-medicalise mental health, including disorders that should be treated with psychological treatments. [51]

Overall, we have a situation in Spain, and in Cantabria in particular, in which there is a health problem with a high prevalence, which causes significant costs for the affected person, their relatives and for society as a whole. However, despite the existence of appropriate psychological therapies recommended by the reference clinical guidelines as the treatment of choice for the most prevalent mental disorders (i.e. those based on CBT), they are hardly used at the level of care through which the health system is accessed and where most of these health problems are treated: PC. Although there are some initiatives worldwide to facilitate access to psychological treatments, in Spain there are no rigorous methodological studies to prove their cost-effectiveness in the PC context. Particularly relevant is the study of treatments that allow their application in formats and approaches that can be easily disseminated in health centres, as is the case of short-term transdiagnostic group treatments.

**Primary purpose:**

Test cost-effectiveness and cost-utility of transdiagnostic cognitive-behavioural treatment versus relaxation therapy in Cantabria Primary Care Centres at posttreatment and 12-month follow-up.

**METHODOLOGY**

**Description of the type of trial**

A prospective, single-blind, randomized controlled trial will be carried out in two PC health centers in Cantabria (Camargo Costa and Camargo Interior). Patients attending their PC center with possible common mental disorders (related to anxiety, depression or somatization) not susceptible to be referred to specialized care according to the criteria of their referring physician in the health center, will be invited to participate in the study, as well as to sign the informed consent form. Those who accept will be assessed before being assigned to groups, so the assessors will be blinded to treatment assignment at all times. Cases who have completed testing will be randomly assigned to the two treatment conditions: (i) control group with treatment using Bernstein and Borkovec's progressive muscle relaxation techniques together with treatment as usual by their GP; or (ii) experimental group with manualized group treatment based on CBT plus treatment as usual by their GP.

Patients assigned to both groups will receive 7 sessions of 1.5 hours duration, in subgroups of approximately 10-12 persons, spread over 12 weeks, with a higher temporal frequency at the beginning of the treatment (one session per week) and progressive extension of the time interval between sessions as the treatment progresses.

**Sample size:**

The study will recruit 300 participants (150 for each arm).

**Name of each arm:**

| Arms | Assigned Interventions |
| --- | --- |
| Experimental: transdiagnostic cognitive-behavioural therapy (TD-CBT)  Transdiagnostic group cognitive-behavioural therapy: The psychological interventions will be manualized. Patients assigned to the experimental group will receive 7 sessions (1.5 hr/session) in groups of approximately 8-10 individuals over a 12-week period. | Behavioural: Transdiagnostic cognitive-behavioural therapy (TD-CBT). |
| Active Comparator: Relaxation therapy  The control group will receive a PMR group intervention, based on the Bernstein and Borkoveck procedure. Patients will receive 7 sessions (1.5 hr/session) in groups of 8-10 individuals over a 12-week period. | Bernstein and Borkovec progressive muscle relaxation (PMR) |

**Recruitment process of participants**

In a first step, GP, as they usually do among those patients who would not be referred to specialised care, will recruit potential candidates with a common mental disorder (anxiety disorders, mild or moderate depression, somatoform disorders) as the main reason for consultation (Phase 1), then psychologists trained with the appropriate tools for the detection of mental disorders will make a correct diagnosis of those disorders of mild and moderate intensity to be treated in PC according to the previously established inclusion and exclusion criteria (Phase 2). If the criteria are met, random allocation to groups will be carried out by blinded research staff, not involved in the assessment or treatment. In the case of not meeting the criteria, the subject would be discarded, recommending consultation with their GP or, if necessary, referral to the Mental Health Centre of reference for their area.

In the post-treatment phase, a psychologist who had not participated in the psychological treatment group would carry out the evaluation phase with the same tests used in the pre-treatment evaluation. This researcher would be blind to the branch of the study to which the assessed subject belongs. The patient will be asked not to disclose which group he/she participated in at any time during the study.

**Ethical considerations**

The proposed research respects and will comply with current legislation and other regulatory standards relevant to the project in the field of ethics, including the fundamental principles of the Declaration of Helsinki, the Council of Europe Convention on Human Rights and Biomedicine, the UNESCO Universal Declaration on the Human Genome and Human Rights, and the Convention for the Protection of Human Rights and Dignity of the Human Being with regard to the Application of Biology and Medicine.

This study will be developed according to the following criteria:

- Briefly explain the ethical principles justifying the research in accordance with international and national regulations.

- Clearly state the risks and safety assurances provided to participants.

- Establish that the research will be carried out when authorisation is obtained from: the legal representative of the research institution and the institution where the research will be carried out; the Informed Consent of the participants; and the approval of the project by the Research Ethics Committee of the institution.

**Inclusion/Exclusion criteria**:

Inclusion criteria:

- Patients aged 18 to 65, inclusive, who present to the primary care centre seeking treatment for anxiety, depressive or somatic symptoms.

- Scores above the predetermined cut-off points on the GAD-7 (>= 10), the PHQ-9 (>= 10) or the PHQ-15 (>=10 plus a score of 2 in three or more somatic symptoms).

- Agreement to participate in the study, with written informed consent.

Exclusion criteria:

- Major depressive disorder (PHQ-9> 24) and/or severe disability (SDS > 25) will be interviewed by a clinician for the presence of any severe mental disorder, including autism spectrum disorders, bipolar disorder, schizophrenia, anorexia nervosa, substance dependence, personality disorder.

- Presence of severe or recent suicide attempts

- Presence of intellectual disability (IQ < 70).

- Be receiving psychological treatment or any type of specialized care related to mental health.

- Insufficient Spanish language skills

**Outcome Measures**

Primary Outcome Measures:

- Change in cost-effectiveness data [Time Frame: Baseline, immediately after the intervention, and 12-month follow-up]

Cost-effectiveness results will be calculated by the ICER, defined as the difference in mean costs between interventions divided by the difference in their effectiveness according to the symptom questionnaires’ mean scores.

The healthcare data collected will be used for cost calculations. To calculate healthcare-related costs, an ad hoc questionnaire will be used to collect emotional disorder-related healthcare data (public and private healthcare consultations, accidents, medical tests, and sick leaves in the past 3 months; psychotropic drugs or other medication, and their posology).

- Change in cost-utility data [Time Frame: Baseline, immediately after the intervention, and 12-month follow-up]

Cost-utility will be measured through the healthcare data collected above and the European Quality of Life Scale (EuroQoL, EQ) [71], calculating the QALYS and the ICURs, defined as the difference in mean cost divided by the difference in mean QALYs. The Spanish version of the 5-domain, 5-level EuroQol (EQ-5D-5L) [72,73] will be used to assess health status in five dimensions (mobility, self-care, daily activities, pain/unease, and anxiety/depression) with five levels of severity (no problems, slight problems, moderate problems, severe problems, and either extreme problems or unable to perform activity). The scores obtained in the different dimensions can be combined to create a 5-digit code describing the patient's state of health, being able to stablish up to 3125 different combinations, and therefore, different possible health states. This system was converted into a utility score using the value set for Spain from the EQ-5D-5L Crosswalk Index Value downloaded from the EuroQol website (<https://euroqol.org/euroqol/>).

Secondary Outcome Measures:

- Change in depressive symptoms: Patient Health Questionnaire - 9 item (PHQ-9) [Time Frame: Baseline, immediately after the intervention, and 12-month follow-up]

The PHQ-9 [54] is the depression module of the PHQ [74, 75] that scores the 9 DSM-IV depression criteria in the last two weeks. Is a nine item, self-report scale that ranges from 0 to 27 (higher scores means a worse outcome).

- Change in anxiety symptoms: Generalized Anxiety Disorder - 7 item (GAD-7) [Time Frame: Baseline, immediately after the intervention, and 12-month follow-up]

The GAD-7 [56] assesses common anxiety symptoms in the last two weeks. It is composed of seven self-report items ranging from 0 to 21 points. Higher scores means a greater presence of anxiety symptoms.

- Change in somatic symptoms: Patient Health Questionnaire - 15 item (PHQ-15) [Time Frame: Baseline, immediately after the intervention, and 12-month follow-up]

The PHQ-15 [58] is the somatization module of the PHQ and scores symptoms present in the past four weeks. The scale is composed of fifteen self-report items, ranging from 0 to 30. Higher scores means a worse outcome.

- Change in functioning: Sheehan Disability Scale (SDS) [Time Frame: Baseline, immediately after the intervention, and 12-month follow-up]

The SDS [59] is a five item self-reported scale composed of three main domains (work, family and social functioning) and two optional items (perceived stress and perceived social support). It ranges from 0 to 50, with higher scores indicating a worse outcome.

- Change in treatment satisfaction [Time Frame: Immediately after the intervention and 12-month follow-up]

Posttreatment and 12-month follow-up assessments will also collect an additional question about treatment satisfaction, through a Likert-type question, ranging from 0 to 10.

Other mechanistic outcome measures:

- Change in rumination: Ruminative Responses Scales (brooding subscale) (RRS-B) [Time Frame: Baseline, immediately after the intervention, and 12-month follow-up]

The RRS-B [76] is composed of five self-reported items, ranging from 5 to 20. Higher scores means a worse outcome.

- Change in worry: Penn State Worry Questionnaire – Abbreviated (PSWQ-A) [Time Frame: Baseline, immediately after the intervention, and 12-month follow-up]

The PSWQ-A [62] measures the pathological worry as an uncontrollable and general state. The scale is composed of eight self-reported items, ranging from 5 to 40. Higher scores means a worse outcome.

- Change in attentional and interpretative biases: Inventory of Cognitive Activity in Anxiety Disorders (IACTA) [Time Frame: Baseline, immediately after the intervention, and 12-month follow-up]

The IACTA was originally developed by Cano-Vindel [63]. It includes subscales that assess distortions according to Eysenck's four-factor theory [77]. The scale is composed of five self-reported items, ranging from 0 to 20. Higher scores means a worse outcome.

- Change in emotion regulation: Cognitive Emotion Regulation Questionnaire (CERQ) [Time Frame: Baseline, immediately after the intervention, and 12-month follow-up]

The CERQ-36 [78] was developed for measuring the specific cognitive emotion regulation strategies that a person uses to face a stressful event (self-blame, acceptance, rumination, positive refocus, refocus on planning, positive reappraisal, putting into perspective, catastrophizing or blaming others). It scores from 1 ("almost never") to 5 ("almost always") how often the participant thinks as described. The 27-item shortened version will be used [79]. Each cognitive strategy is assessed by means of three items, ranging from 3 to 15. Higher scores means a greater use of the strategy.

- Change in metacognitive beliefs: Metacognitions Questionnaire (negative beliefs subscale) (MCQ) [Time Frame: Baseline, immediately after the intervention, and 12-month follow-up]

The MCQ-NB [65] is a short form of the original MCQ [80], which measures the beliefs about the own thinking processes. The scale is composed of six self-reported items, ranging from 6 to 24. Higher scores means a worse outcome.

- Change in Cognitive Distortions in Emotional Disorders (CDTE) [Time Frame: Baseline, immediately after the intervention, and 12-month follow-up]

The CDTE [81] measures the frequency of certain cognitive biases. It includes sixteen self-reported items that measure the presence of four factors: sustained attention bias, divided attention bias, magnification interpretational bias, and catastrophization interpretational bias. It ranges from 0 to 4. Higher scores means a greater presence of the cognitive bias.

- Change in alliance: Working Alliance Inventory Patient Form (WAI-P) and Group Session Rating Scale (GSRS) [Time Frame: In therapy sessions number 1, 4 and 7]

The WAI-P [69] is a thirty-six self-report scale that measure perceived therapeutic alliance. It ranges from 36 to 252 with higher scores indicating better alliance between patient and clinical professional.

The GSRS [70] is a four item self-reported scale that assess alliance to the group. It ranges from 0 to 40, with higher scores indicating better alliance between the patient and the group of therapy.

**The transdiagnostic, group psychological treatment of the experimental group.**

The psychological intervention offered (which will be applied by psychologists) will be protocolised (with a Therapist's Manual) and described in a document addressed to the patient. The patients assigned to the experimental group will receive 7 sessions of 1.5 hours duration in groups of approximately 10 people, spread over 24 weeks, with a greater temporal frequency at the beginning of the treatment (one session per week) and a progressive extension of the time interval between sessions as the treatment progresses. The treatment protocol will be adjusted to the following contents:

Sessions 1: This group will start the psychological treatment with psychoeducational training, in order to provide participants with concrete information (oral and written), as well as information resources (e.g., books, website), on stress, emotions, anxiety, anger, depression, the role of cognitive processes and biases on emotion, emotional learning, emotion regulation, relationships between emotion and behaviour, etc., trying to adapt the contents to the needs of the participants. In all sessions, patients will be motivated to take an active role in the treatment and to carry out the corresponding daily homework.

Session 2: Relaxation techniques will be introduced, with a mixture of Bernstein and Borkovec's progressive muscle relaxation training, abdominal breathing and imagery training. The principles of these exercises, the intended objectives and how to perform the training will be explained. In addition, patients will be asked to practice these exercises daily at home with an audio recording that will be provided, lasting approximately 30 minutes. In order to develop motivation and the habit of practising this task, patients will be asked to make a graphic self-recording of their daily evaluation of the degree of relaxation experienced after the practice, throughout the 24 weeks of the intervention.

Sessions 3-4: These sessions will be devoted to practice in cognitive restructuring and emotional self-regulation training. [66, 67] First, they will be instructed, in a general way, on what emotions are, how they manifest (i.e., at a cognitive-subjective, physiological and behavioural level) anxiety, fear or sadness; as well as in which situations these responses occur, which reactions help to improve adaptation to the environment or which do not, and which strategies are appropriate and which are not to regulate these reactions. It will also be explained how some emotional disorders can easily develop (e.g., when stress increases, uncontrolled anxiety reactions are triggered, too much importance is given to a physiological response that has no voluntary control and attention is focused on the physical sensations it produces). By means of case presentations, it will be shown how it is possible to detect and change distorted thoughts, irrational beliefs, threatening appraisals [68], cognitive biases [52], emotion regulation strategies, etc., which are associated with intense negative emotional states of anxiety, anger or other negative emotions in everyday life. Afterwards, we will work with concrete samples of emotional events experienced by patients in the last week and show them how they should approach these events from a new cognitive-emotional perspective that will help them better understand their errors and biases in emotion regulation, and how they can restore emotional self-regulation through changes in information processing (e.g., encouraging neutral information processing), encouraging neutral or positive information processing, using distraction, reducing the importance and magnification of the threat or loss, reducing ruminative processes, worry and biases of attention focused on the threat or loss, increasing positive self-instructions and perceived self-efficacy, etc.).

Sessions 5-6: These will focus on behavioural techniques and will be aimed at patients learning or relearning and regaining self-management of situations, emotions and behaviours that they had failed to manage correctly in their daily lives. [66,67] This intervention should include a treatment package composed of several psychological techniques (e.g., exposure) that are initiated in the group sessions and should be practised daily at home (in addition to relaxation and self-help book): self-observation, stimulus control, reinforced behavioural training, exposure without safety behaviours, coping skills training, etc. Group sessions will review recently experienced emotional events, reinforce successes, correct errors and continue to encourage decreasing cognitive biases (e.g., attention, interpretation, memory, attribution) that trigger emotional reactions that alter behaviour by providing more homework assignments that involve rehearsal, practice, reinforcement, correction, progressive exposure, etc.

Session 7: This will include relapse prevention, pointing out that relapses do not mean a return to the beginning, a total loss of therapeutic gains, nor a failure, but a new challenge to be overcome and an opportunity to learn more and consolidate what has been learned. To this end, any relapses that may have occurred should be analysed, indicating how to proceed to overcome the difficulties and learn from such setbacks. The session will be completed with a general review of all that has been learned and a generalisation to similar problems that may arise in the future, always emphasising that in order to overcome these difficulties, technical tools are now available, as well as new coping skills (which continue to be developed through practice with these techniques), which have proved useful in overcoming the problems that have already been solved. It will be recommended that these skills be used to cope with future life stress and not the old habits that led to the development of their emotional problems.

**Treatment using relaxation techniques**

The treatment using Bernstein and Borkovec's progressive muscle relaxation technique will be applied by psychologists. Patients assigned to the control group, as in the experimental group, will receive 7 sessions of 1.5 hours duration in groups of approximately 10 people, spread over 24 weeks, with a greater temporal frequency at the beginning of the treatment (one session per week) and a progressive extension of the time interval between sessions as the treatment progresses.

Both groups, experimental and control, together with the treatments described above, will follow the usual treatment by their GP, according to their usual procedure, typically pharmacological treatment and unstructured support. The number of consultations received during the period of participation in the study will be taken into account.

**Statistical analysis**

Both parallel groups will be tested for equivalence on pre-treatment measures. In the efficacy analysis, 2x2 (group x treatment) intergroup-intragroup ANOVAs will be carried out on the dependent measures: 1) symptoms of anxiety, depression and somatisation; 2) cognitive-emotional factors associated with symptoms; 3) occupational, family and social dysfunction; 4) quality of life; 5) satisfaction with care received; 6) cost-effectiveness analysis (e.g., frequentation, drug consumption). The effect size of both groups on the different dependent variables will be calculated, as well as their precision (95% confidence intervals), taking into account the number of sessions received. The percentages of patients in both groups who have decreased their clinical symptom scores by 50% and by one standard deviation, as well as the percentages of probable cases with mental disorders (anxiety, depressive and somatoform) before and after treatment, according to the cut-off points and decision rules for diagnosis, will also be calculated. The primary analyses will follow the "intention-to-treat" principle, so that all individuals who have entered the study will be considered in their corresponding group, even if they have not complied with their protocol. This will allow the objective of randomisation to be maintained until the end of the study, i.e. to reduce the likelihood of biasing the results.

The cost-effectiveness analysis will be carried out by calculating the increase in cost-effectiveness ratios, defined as the difference between the mean costs divided by the increase in effectiveness between the different therapeutic alternatives compared (ICER).

**CHRONOGRAM AND WORK PLAN**

Months 1: Involvement of professionals in the project and training of clinical psychologists in group psychological treatment through a workshop conducted by experts from the Spanish Society for the Study of Anxiety and Stress (SEAS), under the supervision of Prof. Antonio Cano Vindel.

Months 2-11: Recruitment of patients (progressive over 10 months) by GP in Health Centres, randomisation, implementation of therapy groups and evaluations by clinical psychologists, data collection.

Months 10-12: Completion of data collection and analysis and generation of draft articles. Dissemination of preliminary project data at national and international conferences.

**APPLICABILITY AND USEFULNESS OF THE RESULTS AND ABILITY TO BE PROTECTED AND TRANSFERRED TO THE MARKET**

The planned clinical trial will provide clear evidence that, compared to treatment through relaxation, the incorporation of a group treatment for mental disorders commonly treated in Health Centres helps to reduce anxious, depressive and somatic symptomatology, as well as a reduction in the costs (direct and indirect) associated with anxiety, mood and somatoform disorders (Main hypothesis). PsicAP-Costs2 allows an immediate transfer of the results to the clinical context by providing the first experience in our context of a manualised transdiagnostic group treatment of short duration (7 sessions), which makes it easily applicable and generalizable to health centres in Cantabria and the rest of the NHS. The expected economic benefit in the reduction of psychopharmacological treatment costs and through the reduction of expenditure on sick leave and loss of productivity, which is a priority objective of the SCS in the management of TI associated with common mental disorders (Mental Health Plan of Cantabria 2015-2019), should make the dissemination among health professionals of this type of protocolised treatment, low cost and short duration, easily integrated into the routine care offered in Health Centres, profitable.

**Locations**

Spain

Centro Sanitario “Camargo Costa” **Recruiting**

C. Julio de Pablo, 0, 39600, Maliaño, Cantabria

Centro Sanitario "Camargo Interior" **Recruiting**

Av. de Bilbao, s/n, 39600 Maliaño, Cantabria

Centro Sanitario "Sardinero" **Recruiting**

C. Alcalde Vega Lamera, 6, 39005 Santander, Cantabria

Centro Sanitario “Dávila” **Recruiting**

P.º del Gral. Dávila, 71, 39006 Santander, Cantabria

**Sponsors and Collaborators**

Instituto de Investigación Sanitaria Valdecilla (IDIVAL)

Agencia Estatal de Investigación

**BIBLIOGRAPHY**

1. Haro JM, Palacín C, Vilagut G, Martínez M, Bernal M, Luque I, Codony M, DolzM, Alonso J; Grupo ESEMeD-España. Prevalencia de los trastornos mentales y factores asociados: resultados del estudio ESEMeD-España. Med Clin (Barc). 2006 Apr 1;126(12):445-51.

2. Vázquez-Barquero JL, Díez-Manrique JF, Peña C, Aldama J, Samaniego Rodríguez C, Menéndez Arango J, Mirapeix C. A community mental health survey in Cantabria: a general description of morbidity. Psychol Med. 1987 Feb;17(1):227-41.

3. Haro JM, Pinto-Meza A, Serrano-Blanco A. Epidemiología de los trastornos mentales en Atención Primaria. En Psiquiatría en Atención Primaria. José Luis Vázquez-Barquero (Ed.). Madrid: Grupo Aula Médica, S.L. 2007:41-59.

4. Layard R, Clark DM. Thrive: The Power of Evidence-Based Psychological Therapies. London: Penguin. 2014.

5. Wittchen HU, Jacobi F, Rehm J, Gustavsson A, Svensson M, Jönson B, Olesen J, Allgulander C, Alonso J, Faravelli C et al. The size and burden of mental disorders and other disorders of the brain in Europe 2010. European Neuropsychopharmacology. 2011;21:655-679.

6. Gobierno de Cantabria. Plan de Salud Mental de Cantabria 2014-2019. Santander: Consejería de Sanidad y Servicios Sociales. 2014.

7. Calvo Bonacho E. Duración de la incapacidad temporal asociada a diferentes patologías en trabajadores españoles: discusión acerca de la ITCC en los trastornos mentales. Madrid. 2010. Disponible en http://www.seg-social.es/prdi00/groups/public/documents/binario/146666.pdf. Fecha acceso: 8.02.2016.

8. Gustavsson A, Svensson M, Jacobi F, Allgulander C, Alonso J, Beghi E, Dodel R et al. Cost of disorders of the brain in Europe 2010. European Neuropsychopharmacology. 2011;21:718-779.

9. Parés-Badell O, Barbaglia G, Jerinic P, Gustavsson A, Salvador-Carulla L, Alonso J. Cost of disorders of the brain in Spain. PLoS One.2014;18:9(8):e105471.

10. Melek S, Norris D. Chronic Conditions and Comorbid Psychological Disorders. Seattle: Milliman. 2008.

11. De Hert M, Correll CU, Bobes J, Cetkovich-Bakmas M, Cohen D, Asai I, Detraux J, Gautam S, Möller HJ, Ndetei DM, Newcomer JW, Uwakwe R, Leucht S. Physical illness in patients with severe mental disorders. I. Prevalence, impact of medications and disparities in health care. World Psychiatry.2011;10(1):52-77.

12. Zuidersma M, Ormel J, Conradi HJ, de Jonge P. An increase in depressive symptoms after myocardial infarction predicts new cardiac events irrespective of depressive symptoms before myocardial infarction. Psychol Med.2012;42:683-93.

13. Joukamaa M, Heliövaara M, Knekt P, Aromaa A, Raitasalo R, Lehtinen V. Mental disorders and cause-specific mortality. Br J Psychiatry. 2001 Dec;179:498-502.

14. Nordentoft M, Wahlbeck K, Hällgren J, Westman J, Osby U, Alinaghizadeh H, Gissler M, Laursen TM. Excess mortality, causes of death and life expectancy in 270,770 patients with recent onset of mental disorders in Denmark, Finland and Sweden. PLoS One.2013; 8(1):e55176.

15. Cuijpers P, Smit F. Excess mortality in depression: a meta-analysis of community studies. J Affect Disord. 2002 Dec;72(3):227-36.

16. Harris EC, Barraclough B. Suicide as an outcome for mental disorders. A meta-analysis. Br J Psychiatry.1997 Mar; 170: 205-28.

17. Arsenault-Lapierre G, Kim C, Turecki G. Psychiatric diagnoses and 3275 suicides: a metanalysis. BMC Psychiatry.2004;4:37.

18. Instituto Nacional de Estadística. Defunciones según la causa de muerte Madrid: Instituto Nacional de Estadística. 2013 [Documento Internet, Acceso 08/02/2016]. Disponible en: www.ine.es.

19. Luoma JB, Martin CE, Pearson JL. Contact with mental health and primary care providers before suicide: a review of the evidence. Am J Psychiatry. 2002;159:909-16.

20. Wittchen H.-U, Jacobi F. Size and burden of mental disorders in Europe_a critical review and appraisal of 27 studies. Eur. Neuropsychopharmacol. 2005;15(4):357-376.

21. Kessler RC, Ustun B. The WHO World Mental Health Surveys: Global Perspectives on the Epidemiology of Mental Disorders. New York: Cambridge University Press .2008.

22. Butler AC, Chapman JE, Forman EM, Beck AT. The empirical status of cognitive-behavioral therapy: a review of meta-analyses. Clin Psychol Rev. 2006;26:17-31.

23. Cuijpers P, Berking M, Andersson G, Quigley L, Kleiboer A, Dobson KS. A meta-analysis of cognitive-behavioural therapy for adult depression, alone and in comparison with other treatments. Can J Psychiatry. 2013 Jul;58(7):376-85.

24. Hollon SD, Stewart MO, Strunk DR. Enduring effects for cognitive behaviour therapy in the treatment of depression and anxiety. Annu Rev Psychol. 2006;57:285–315.

25. Spielmans GI, Berman MI, Usitalo AN. Psychotherapy versus second-generation antidepressants in the treatment of depression: a meta-analysis. J Nerv Ment Dis. 2011;199:142–9.

26. Van Orden M, Hoffman T, Haffmans J, Spinhoven P, YHoencamp E. Collaborative Mental Health Care versus Care as Usual in a Primary Care Setting: a randomised controlled trial. Psychiatr Serv. 2009;60(1):74-9.

27. Twomey C, O'Reilly G, Byrne M. Effectiveness of cognitive behavioural therapy for anxiety and depression in primary care: a meta-analysis. Fam Pract. 2015 Feb;32(1):3-15.

28. Linde et al. Effectiveness of Psychological Treatments for Depressive Disorders in Primary Care: Systematic Review and Meta-Analysis. 2015.

29. Feng C, Chu H, Chen C, Chang Y, Chang Y, Chen T, . . . & Cou K. The effect of cognitive behavioral group therapy for depression: A metaanalysis 2000-2010. World Views on Evidence-Based Nursing. 2011; First Quarter: 2-16.

30. Huntley A, Araya R, Salisbury C. Group psychological therapies for depression in the community: Systematic review and meta-analysis. The British Journal of Psychiatry. 2012; 200: 184-190.

31. González González S, Fernández Rodríguez C, Pérez Rodríguez J, Amigo I. Prevención secundaria de la depresión en atención primaria. Psicothema. 2006 Aug;18(3):471-7.

32. Segarra, G., Farriols, N., Palma, S., Segura, J., & Castell, R. (). Tratamiento psicológico grupal para los trastornos de ansiedad en el ámbito de la salud pública. Ansiedad & Estrés. 2011;17,185-197.

33. National Institute for Health and Clinical Excellence (NICE). Common Mental Health Disorders: Identification and Pathways to Care. NICE Clinical Guideline 123. 2011. Accesible en www.nice.org.uk/CG123 [NICE guideline].

34. McHugh RK, Whitton SW, Peckham AD, Welge JA, Otto MW. Patient preference for psychological vs pharmacologic treatment of psychiatric disorders: a meta-analytic review. J Clin Psychiatry. 2013 Jun; 74(6): 595-602.

35. Swift JK, Greenberg RP, Tompkins KA, Parkin SR. Treatment refusal and premature termination in psychotherapy, pharmacotherapy, and their combination: A meta-analysis of head-to-head comparisons. Psychotherapy (Chic). 2017;54:47-57.

36. Swift JK, Callahan JL, Vollmer BM. Preferences. J Clin Psychol. 2011 Feb; 67(2):155-65.

37. Mergl R, Henkel V, Allgaier AK, Kramer D, Hautzinger M, Kohnen R, Coyne J,Hegerl U. Are treatment preferences relevant in response to serotonergic antidepressants and cognitive-behavioral therapy in depressed primary care patients? Results from a randomized controlled trial including a patients' choice arm. Psychother Psychosom. 2011; 80(1): 39-47.

38. Autonell J, Vila F, Pinto-Meza A, Vilagut G, Codony M, Almansa J, Muñoz PE, Torres JV, Alonso J, Haro JM. Prevalencia-año de la comorbilidad de los trastornos mentales y factores de riesgo sociodemográficos asociados en la población general de España. Resultados del estudio ESEMeD-España. Actas Esp Psiquiatr. 2007; 35 Suppl 2:4-11.

39. Cordero-Andrés, P., González-Blanch, C., Umaran-Alfageme, O., Muñoz-Navarro, R., Ruíz-Rodríguez, P., Medrano, L.A., et al. Tratamiento psicológico de los trastornos emocionales en atención primaria: fundamentos teóricos y empíricos del estudio PsicAP. Ansiedad y Estrés, 2017; 23: 91-98.

40. Brown TA, Barlow DH. A proposal for a dimensional classification system based on the shared features of the DSM-IV anxiety and mood disorders: implications for assessment and treatment. Psychol Assess. 2009 Sep;21(3):256-71.

41. Tortella-Feliu M, Aguayo B, Sesé A, Morillas-Romero A, Balle M, Gelabert JM, Bornas X, Llabrés J. Effects of temperament and emotion regulation styles in determining negative emotional states. Actas Esp Psiquiatr. 2012; 40(6):315-22.

42. Barlow, D. H., Farchione, T. J., Fairholme, C. P., Ellard, K. K., Boisseau, C. L., Allen, L. B., Ehrenreich-May, J. (2015) Protocolo unificado para el tratamiento transdiagnóstico de los trastornos emocionales. Madrid: Alianza Editorial.

43. Porto PR, Oliveira L, Mari J, Volchan E, Figueira I, Ventura P. Does cognitive behavioral therapy change the brain? A systematic review of neuroimaging in anxiety disorders. J Neuropsychiatry Clin Neurosci. 2009;21(2):114-25.

44. National Institute for Health and Clinical Excellence (NICE). Putting guidance into practice. In. UK: NICE. Acceso 22-July-2011, de http://www.nice.org.uk/usingguidance/using_guidance.jsp; 2010.

45. Clark DM, Layard R, Smithies R, Richards DA, Suckling R, Wright B. Improving access to psychological therapy: Initial evaluation of two UK demonstration sites. Behav Res Ther. 2009; 47(11): 910-20.

46. Richards DA, Suckling R. Improving access to psychological therapies: phase IV prospective cohort study. Br J Clin Psychol. 2009; 48(Pt 4): 377-96.

47. Health and Social Care Information Centre Psychological Therapies. Annual Report on the Use of IAPT Services: England. HSCIC. 2014.

48. Codony M, Alonso J, Almansa J, Vilagut G, Domingo A, Pinto-Meza A, et al. Utilización de los servicios de salud mental en la población general española. Resultados del estudio ESEMeD-Espana. [Mental health care use in the Spanish general populations. Results of the ESEMeD-Spain study]. Actas Esp Psiquiatr. 2007; 35 (Suppl 2): 21-8.

49. Kessler RC, Aguilar-Gaxiola S, Alonso J, Chatterji S, Lee S, Ormel J, et al. The global burden of mental disorders: an update from the WHO World Mental Health (WMH) surveys. Epidemiol Psichiatr Soc. 2009; 18(1):23-33.

50. Fernández A, Haro JM, Codony M, Vilagut G, Martinez-Alonso M, Autonell J, et al. Treatment adequacy of anxiety and depressive disorders: primary versus specialised care in Spain. J Affect Disord. 2006; 96(1-2):9-20.

51. SNS – Sistema Nacional de Salud. Estrategia en Salud Mental, 2011. [Accesso 13/02/2016] http://www.msssi.gob.es/organizacion/sns/planCalidadSNS/docs/saludmental/SaludMental2009-2013.pdf.

52. Eysenck MW, Derakshan N. Un marco teórico cognitivo para los trastornos de ansiedad. Ansiedad y Estrés. 1997; 3(2-3):121-134.

53. Kroenke K, Spitzer RL, Williams JB, Lowe B. The Patient Health Questionnaire Somatic, Anxiety, and Depressive Symptom Scales: a systematic review. Gen Hosp Psychiatry. 2010;32(4):345-59.

54. Kroenke K, Spitzer RL, Williams JB. The PHQ-9: validity of a brief depression severity measure. J Gen Intern Med. 2001;16(9):606-13.

55. Kroenke K, Spitzer RL, Williams JB, Lowe B. An ultra-brief screening scale for anxiety and depression: the PHQ-4. Psychosomatics. 2009;50(6):613-21.

56. Spitzer RL, Kroenke K, Williams JB, Lowe B. A brief measure for assessing generalized anxiety disorder: the GAD-7. Arch Intern Med. 2006; 166(10): 1092-7.

57. Wittkampf KA, Baas KD, van Weert HC, Lucassen P, Schene AH. The psychometric properties of the panic disorder module of the Patient Health Questionnaire (PHQ-PD) in high-risk groups in primary care. J Affect Disord. 2011; 130(1-2): 260-7.

58. Kroenke K, Spitzer RL, Williams JB. The PHQ-15: validity of a new measure for evaluating the severity of somatic symptoms. Psychosom Med. 2002; 64(2): 258-66.

59. Sheehan DV, Harnett-Sheehan K, Raj BA. The measurement of disability. Int Clin Psychopharmacol. 1996; 11(Suppl 3): 89-95.

60. Rocha NS, Power MJ, Bushnell DM, Fleck MP. Cross-Cultural Evaluation of the WHOQOL-BREF Domains in Primary Care Depressed Patients Using Rasch Analysis. Med Decis Making. 2012; 32(1): 41-55.

61. Nolen-Hoeksema S, Morrow J. A prospective study of depression and posttraumatic stress symptoms after a natural disaster: the 1989 Loma Prieta Earthquake. J Pers Soc Psychol. 1991; 61(1): 115-21.

62. Meyer TJ, Miller ML, Metzger RL, Borkovec TD. Development and validation of the Penn State Worry Questionnaire. Behav Res Ther. 1990; 28(6): 487-95.

63. Cano-Vindel A. Inventario de Actividad Cognitiva en los Trastornos de Ansiedad (IACTA). In: Facultad de Psicología. Universidad Complutense de Madrid. No publicado. Madrid. 2001.

64. Dominguez-Lara, S., & Medrano, L. (2016a). Propiedades psicométricas del Cognitive Emotional Regulation Questionnaire (CERQ) en estudiantes universitarios de Lima. Psychologia: Avances en la disciplina, 10(1), 53 – 67.

65. Wells A, Cartwright-Hatton S. A short form of the metacognitions questionnaire: properties of the MCQ-30. Behav Res Ther. 2004; 42(4): 385-96.

66. Cano-Vindel A. Bases teóricas y apoyo empírico de la intervención psicológica sobre los desórdenes emocionales en Atención Primaria. Una actualización. Ansiedad y Estrés. 2011; 17(2-3): 157-184.

67. Cano-Vindel A, Dongil-Collado E, Salguero JM, Wood CM. Intervención cognitivo-conductual en los trastornos de ansiedad: una actualización. Informació Psicològica. 2011; 102: 4-27.

68. Cano-Vindel A. Técnicas cognitivas en el control del estrés. In: Fernández-Abascal EG, Jiménez Sánchez MP, editores. Control del Estrés. Madrid: UNED Ediciones. 2002: 247-271.

69. Andrade-González N, Fernández-Liria A. (2015). Spanish adaptation of the Working Alliance Inventory (WAI): Psychometric properties of the patient and therapist forms (WAI-P and WAI-T). *Anales de Psicología, 31*, 524–533.

70. Duncan, B. L., & Miller, S. D. (2007). The group session rating scale. Jenson Beach, FL: Author.

71. EuroQol Group. EuroQol--a new facility for the measurement of health-related quality of life. Health Policy. 1990 Dec;16(3):199-208.

72. Badia X, Roset M, Montserrat S, Herdman M, Segura A. [The Spanish version of EuroQol: a description and its applications. European Quality of Life scale]. Med Clin (Barc). 1999;112 Suppl 1:79-85. Review. Spanish.

73. van Reenen M, Janssen B, Stolk E, Secnik Boye K, Herdman M, Kennedy-Martin M, et al. EQ-5D-5L User Guide. 3.0. EuroQol Research Foundation; 2019. Available from: <https://euroqol.org/publications/user-guides/>

74. Diez-Quevedo C, Rangil T, Sanchez-Planell L, Kroenke K, Spitzer RL. Validation and utility of the patient health questionnaire in diagnosing mental disorders in 1003 general hospital Spanish inpatients. Psychosom Med. 2001 Jul-Aug;63(4):679-86.

75. Spitzer RL, Kroenke K, Williams JB. Validation and utility of a self-report version of PRIME-MD: the PHQ primary care study. Primary Care Evaluation of Mental Disorders. Patient Health Questionnaire. JAMA. 1999 Nov 10;282(18):1737-44

76. Nolen-Hoeksema S, Morrow J. A prospective study of depression and posttraumatic stress symptoms after a natural disaster: the 1989 Loma Prieta Earthquake. J Pers Soc Psychol. 1991 Jul;61(1):115-21.

77. Eysenck MW. A cognitive approach to trait anxiety. Eur J Pers. 2000 Sep;14(5):463-76

78. Garnefski N, Kraaij V, Spinhoven P. Negative life events, cognitive emotion regulation and emotional problems. Pers Individ Dif. 2001;30(8):1311-27.

79. Holgado-Tello, F. P., Amor, P. J., Lasa-Aristu, A., Domínguez-Sánchez, F. J., & Delgado, B. J. A. D. P. A. o. P. (2018). Two new brief versions of the Cognitive Emotion Regulation Questionnaire and its relationships with depression and anxiety. 34(3), 458-464.

80. Cartwright-Hatton S, Wells A. Beliefs about worry and intrusions: the Meta-Cognitions Questionnaire and its correlates. J Anxiety Disord. 1997 May-Jun;11(3):279-96.

81. The PsicAP Group. Cuestionario de Distorsiones Cognitivas en Trastornos Emocionales (CDTE) [Questionnaire of Cognitive Distortions in Emotional Disorders (CDTE)]. Unpublished
