# Supplementary material for: Cost-effectiveness of transdiagnostic group cognitive behavioural therapy versus group relaxation therapy for emotional disorders in primary care (PsicAP-Costs2): Protocol for a multicentre randomised controlled trial
Source: PLoS One. 2023 Mar 16;18(3):e0283104. doi: 10.1371/journal.pone.0283104 (PMC10019745; doi:10.1371/journal.pone.0283104)
Supplement: S2 File — (DOCX) [file pone.0283104.s004.docx]

**TÍTULO OFICIAL:** COSTE-EFICACIA DEL TRATAMIENTO PSICOLÓGICO GRUPAL TRANSDIAGNÓSTICO VERSUS TRATAMIENTO DE GRUPAL DE RELAJACIÓN PARA TRASTORNOS MENTALES COMUNES EN ATENCIÓN PRIMARIA (PSICAP-COSTS2): ENSAYO CLÍNICO CONTROLADO ALEATORIZADO

**TÍTULO ABREVIADO:** COSTE-EFICACIA DEL TRATAMIENTO PSICOLÓGICO TRANSDIAGNÓSTICO PARA TRASTORNOS EMOCIONALES EN ATENCIÓN PRIMARIA (PSICAP-COSTS2)

**Investigadores**

- César González-Blanch Bosch, PhD.

Centro de Salud Mental, Hospital Universitario Marqués de Valdecilla - IDIVAL, Santander, España.

Facultad de Ciencias de la Salud, Universidad Europea del Atlántico, Santander, España

- Antonio Cano Vindel, PhD.

Facultad de Psicología. Universidad Complutense de Madrid, Madrid, España

**Contactos**

Contacto: César González-Blanch, PhD

Tel: +34-942-202537 Fax: +34-942-203447

E-mail: cesar.gonzalezblanch@scsalud.es

**RESUMEN**

El objetivo de este estudio es probar la coste-eficacia y coste-utilidad de añadir una terapia cognitivo-conductual grupal transdiagnóstica (TD-CBT) al tratamiento habitual (TAU) para los trastornos emocionales en atención primaria. Se realizará un ensayo clínico controlado aleatorizado simple ciego para comparar la terapia grupal TD-CBT más TAU con el grupo de relajación muscular progresiva (PMR) más TAU en personas de 18 a 65 años con trastornos emocionales en cuatro centros de atención primaria ubicados en Cantabria, España. El estudio tendrá una perspectiva social. Se realizarán evaluaciones psicológicas al inicio, después del tratamiento y a los 12 meses de seguimiento. Las evaluaciones incluirán medidas de síntomas clínicos (ansiedad, depresión y/o somáticos), funcionamiento, factores cognitivo-emocionales (rumiación, preocupación patológica, sesgos atencionales e interpretativos, estrategias de regulación emocional y creencias metacognitivas) y satisfacción con el trato recibido. Los datos sobre el uso de los servicios de salud, incluida la medicación y los días de ausencia del trabajo, se recopilarán de los registros médicos electrónicos. Los resultados primarios son los ratio de coste eficacia incremental (ICER) basados ​​en la diferencia en los costos medios y la efectividad entre las intervenciones y los Ratios coste-utilidad incrementales (ICUR) basados ​​en la calidad de vida relacionada con la salud después del tratamiento y a los 12 meses de seguimiento. Las medidas de resultado secundarias incluyen síntomas clínicos, calidad de vida, funcionamiento y satisfacción con el tratamiento. Se utilizará un muestreo bootstrap para evaluar la incertidumbre de los resultados. También se realizarán análisis secundarios de moderación y mediación. Además, en las sesiones número 1, 4 y 7 de ambos brazos de tratamiento, se administrarán dos cuestionarios que recogen la alianza terapéutica y la satisfacción grupal. La hipótesis principal del estudio es que añadir TD-CBT a TAU en atención primaria será más coste-eficaz que TAU más PMR. Además, estas ganancias se mantendrán en el seguimiento de 12 meses. Si tiene éxito, la difusión de un tratamiento coste-eficaz puede ayudar a superar los problemas de acceso al tratamiento psicológico de los trastornos emocionales en el contexto de una demanda creciente de atención de la salud mental en la atención primaria.

**Patologías:**

Trastornos emocionales (depresión, ansiedad y trastornos somatomorfos)

**Palabras clave:**

terapia transdiagnóstica

relajación muscular progresiva

trastornos emocionales

atención primaria

análisis de coste-efectividad

análisis de coste-utilidad

tratamientos psicológicos breves

tratamiento cognitivo-conductual

ensayo controlado aleatorizado

depresión

ansiedad

trastornos somatomorfos

**ANTECEDENTES Y ESTADO ACTUAL DEL TEMA Y BIBLIOGRAFÍA**

**La dimensión de los trastornos mentales**

Estudios epidemiológicos en España apuntan que alrededor de 1 de cada 5 personas presentarán un trastorno mental a lo largo de su vida [1] y un 8.4% durante los últimos 12 meses. Los trastornos de ansiedad (6.2%) y los trastornos del estado de ánimo (4.4%) son los más frecuentes en los últimos 12 meses [1]. En Cantabria, la prevalencia puntual en algunos estudios epidemiológicos se eleva a 14.7%. [2] En el caso de los centros de salud de Atención de Atención Primaria (AP), se puede estimar, a partir de los distintos estudios epidemiológicos en nuestro entorno, que alrededor de un 30-35% de las personas que atienden presentan un trastorno mental. En este nivel asistencial los trastornos de ansiedad son los más frecuentes, seguidos por los depresivos. [3]

**El coste de los trastornos mentales comunes**

En los países desarrollados, el 38% de las enfermedes es un trastorno mental [4], afectando especialmente a las personas en edad de trabajar en las que representa el 50% del total de las enfermedades. Estudios recientes realizados en Europa sitúan los trastornos mentales como la primera causa de pérdida de la calidad de vida expresada como Años de Vida Ajustados a Discapacidad (siglas en inglés, DALYs) y como el principal motivo de solicitud de pensión por incapacidad. [5] En España los trastornos mentales son la segunda causa en frecuencia (por detrás de los diagnósticos de origen ósteo-muscular) de los procesos gestionados de incapacidad temporal (IT) por parte de los médicos de AP. Además, según datos de Cantabria, más del 80% de los diagnósticos recogidos en los partes de IT por salud mental corresponden a trastornos “neuróticos” o “menores”. [6] Paradójicamente, los trastornos mentales con mejor pronóstico (esto es, los relacionados con la ansiedad, la depresión y los trastornos adaptativos) son una de las principales fuentes de gasto por prestaciones de IT, al combinar dos características: alta frecuencia y duración muy prolongada. [7]

Los costes económicos de los trastornos mentales se estimaron para 2010 en la Unión Europea en 461.000 millones de euros, excluyendo los costes de las demencias y los trastornos neurológicos; la parte principal de esta cifra corresponde a costes indirectos ligados a la pérdida de productividad y las pensiones por discapacidad. [8] En España, el coste social de los trastornos mentales (excluyendo la demencia y otros trastornos neurológicos) representó 46.000 millones de euros en el 2010, siendo los trastornos relacionados con la ansiedad y la depresión, con más de 10.000 millones cada uno, los que originan las mayores partidas en ese gasto. [9]

Estos costes están subestimados porque no toman en consideración, entre otros, el efecto de la comorbilidad de los trastornos mentales en las personas con enfermedades físicas. Por ejemplo, las personas con enfermedades físicas crónicas (asma, cáncer, EPOC, artritis, hipertensión, diabetes, infartos y problemas cardiacos) con trastornos ansioso-depresivos comórbidos tienen un promedio de gastos médicos mensuales entre 33 y 169% más alto que la media para esas enfermedades, excluyendo los gastos directos de los servicios de salud mental. [10] Por otra parte, los trastornos mentales aumentan el riesgo de aparición de algunas enfermedades físicas. [11] Las personas con depresión tienen un mayor riesgo de desarrollar un problema de corazón; después de un ataque al corazón, cada síntoma depresivo adicional que se desarrolle aumenta el riesgo de otro ataque al corazón en un 15%. [12]

El exceso de mortalidad está bien establecido entre los pacientes con trastornos mentales en comparación con la población general. [13,14,15] En particular, el riesgo de suicidio está aumentado para todos los trastornos mentales, no sólo para los trastornos más graves, sino que los trastornos adaptativos, una categoría diagnóstica residual, quintaesencia de lo leve, tienen un riesgo de suicidio más de 10 veces mayor al de la población general. [16] Las autopsias psicológicas indican que en torno al 90% de los suicidios tienen como motivo principal algún trastornos mental. [17] En España, el suicidio es la causa más probable de muerte para un hombre de entre 20 y 49 años. Tomando las cifras en conjunto, el número de muertes por suicidio anuales, 3.870 personas en 2013, son superiores a las de los accidentes de tráfico, las caídas, los asesinatos o las drogas. [18] Por último, se ha señalado que el contacto con los profesionales de AP en el tiempo previo al suicidio es común; sin embargo, aunque tres de cada cuatro víctimas de suicidio tuvieron contacto con los profesionales de AP dentro del año del suicidio, sólo un tercio de las víctimas había tenido contacto con los servicios de salud mental. [19]

**Los trastornos mentales se tratan poco, tarde y mal**

Una revisión de datos epidemiológicos extraídos de 27 estudios que incluían 150.000 personas de 16 países europeos puso de relieve, además del gran tamaño y gama de alteraciones psicosociales asociados a los trastornos mentales, que éstos tienen una ratio relativamente baja de tratamiento. Sólo 26% de todos los casos con trastornos mentales había tenido consultas formales de atención sanitaria por razones de salud mental en el último mes. [20] Entre los casos tratados con trastornos mentales, había un largo retraso entre inicio del trastorno y el primer contacto para el tratamiento y sólo un pequeño número de pacientes recibió una intervención mínimamente adecuada. [21]

**La eficacia de la terapia cognitivo-conductual**

Varios meta-análisis han demostrado que la TCC es eficaz para los trastornos mentales comunes, tales como los de la ansiedad y la depresión. Se han encontrado tamaños del efecto grandes en trastornos como la depresión unipolar, ansiedad generalizada, trastornos de pánico con o sin agorafobia, fobia social o trastorno de estrés postraumático. [22] En el caso de la depresión, la TCC, la forma de psicoterapia más ampliamente investigada, un meta-análisis reciente encontraba un tamaño del efecto moderado-grande (Hedges g = 0.71), lo que se corresponde con un número necesario para tratar (NNT) de 2.6. [23]

Hay una fuerte evidencia de que la eficacia de la terapia psicológica y la TCC en particular es igual o mayor que los tratamientos farmacológicos para los trastornos afectivos y de ansiedad más comunes, así como tener una mayor efectividad a largo plazo. [22,24-25] En particular, los beneficios de los enfoques psicológicos incluyen una reducción en los síntomas asociados con la ansiedad y la depresión, una disminución en el riesgo de recaídas, la estabilidad del efecto del tratamiento a largo plazo, y altas tasas de recuperación, la prevención de la cronicidad y la disminución de los costes de salud, en términos de las consultas médicas y el uso de fármacos, así como las bajas por enfermedad. [26]

En el caso de la investigación sobre la efectividad de la TCC en el contexto de AP, aunque el número de estudios es menor, un meta-análisis reciente mostró que el tratamiento basado en la TCC para los trastornos depresivos y de ansiedad es más eficaz que no recibir tratamiento (d =0.59) y que el tratamiento habitual en AP (d = 0.48). [27] Hay que destacar que existe evidencia sustancial de que los tratamientos psicológicos con el enfoque de la TCC en pacientes deprimidos en AP tratados con intervenciones de menor intensidad de recursos obtienen resultados similares a los de los tratamientos más intensos. [28] Esto es especialmente relevante a la vista de que el tratamiento grupal basado en la TCC puede ser una modalidad particular rentable. Un meta-análisis de 32 estudios con adultos con depresión encontró que tras la intervención los niveles de depresión se habían reducido significativamente con un tamaño del efecto moderado (g=0.40) comparado con los controles, y estos resultados se mantenían en el seguimiento a los 6 meses. [29] El típico formato en los estudios incluidos era de grupos de 6-10 participantes, con una terapia de 1 hora que se extendía a lo largo de 2-8 semanas. De modo similar, la investigación en 14 estudios con terapias psicológicas grupales en AP (n = 1,217), comparando el tratamiento habitual en AP con la terapia psicológica grupal más el tratamiento habitual, ha mostrado una mejoría clínica con un efecto considerable al finalizar el tratamiento (d = 0.55). [30] La investigación en este campo en España es escasa. Algunos trabajos apuntan la eficacia de intervenciones psicológicas grupales basadas en la TCC en AP para la depresión tanto a corto como a largo plazo [31], y también en el caso de los trastornos de ansiedad. [32] Si bien las debilidades metodológicas de estos estudios obligan a ser cautos con la interpretación de los resultados.

Basándose en los resultados de la extensa investigación sobre los efectos de los tratamientos psicológicos, las guías del *National Institute for Health and Care Excellence* (NICE) recomiendan el uso de los tratamientos psicológicos, particularmente la TCC, en AP antes que los tratamientos farmacológicos para la depresión mayor de intensidad leve y moderada, el trastorno de ansiedad generalizada, el trastorno de estrés postraumático, el obsesivo-compulsivo y el de pánico, esto es, para los trastornos mentales más comunes. [33]

Las guías clínicas hacen hincapié en la conjunción de la evidencia empírica con los valores del paciente y sus preferencias en la selección y aplicación de tratamientos. Los pacientes con trastornos mentales en conjunto muestran una preferencia 3 veces mayor por el tratamiento psicológico que por el farmacológico. [34] Un metanálisis reciente establece que el tratamiento farmacológico es 1,76 más probable de ser rechazado y 1,20 de ser abandonado que la psicoterapia [35] En este sentido, es relevante destacar que las preferencias de los pacientes pueden afectar directamente a los resultados de la terapia, hay una creciente evidencia que indica que proporcionar al paciente su tratamiento preferido está asociado con una mayor adherencia y mejores resultados clínicos. [36] Así, se ha observado que cuando los pacientes de AP con depresión recibían el tratamiento preferido aumentaba significativamente su recuperación medida por la escala de depresión HAMD-17: +2.9 puntos para el tratamiento farmacológico y, en el caso de la TCC grupal, en +8.0 puntos, que debe considerse como clínicamente significativo. [37]

**Un enfoque novedoso para el tratamiento de los trastornos mentales: el modelo transdiagnóstico**

Pese a que la investigación ha demostrado la eficacia de los tratamientos psicológicos para los trastornos mentales comunes, sólo una pequeña parte de los que sufren estos trastornos mentales los reciben. Se han esgrimido varias razones de la infrautilización de los tratamientos psicológicos, como el estigma, la falta de formación de los profesionales en los mismos o, entre otras razones, la multiplicidad de protocolos de tratamiento para distintos trastornos, que suelen tener notable comorbilidad, lo que dificulta la decisión del clínico sobre qué protocolo seguir. Las altas tasas de comorbilidad psíquica, que en algunos trastornos están por encima del 80% [38], la dificultad para delimitar etiologías específicas para los más de 400 trastornos mentales recogidos en los manuales diagnósticos y la cuestionada validez de los mismos es lo que ha dado lugar a nuevas propuestas de clasificación e intervención basadas en modelos transdiagnósticos [39]. Esta perspectiva asume que los distintos trastornos emocionales comparten características importantes y proponen que este solapamiento emerge de vulnerabilidades biológicas y psicológicas comunes que, asociada a factores de estrés psicosocial, puede dar lugar a diferentes manifestaciones de la misma vulnerabilidad. [40] La perspectiva transdiagnóstica enfatiza el papel de la regulación emocional como un concepto clave para entender y tratar los trastornos emocionales. El concepto de regulación emocional se refiere a las estrategias que las personas realizan para modificar las experiencias emocionales que experimentan. Se ha comprobado que las estrategias de regulación emocional inadecuadas como la rumiación y la evitación desempeñan un papel importante en la aparición y mantenimiento de los síntomas de ansiedad y depresión predominantes en los trastornos mentales comunes. [41] El modelo transdiagnóstico aprovecha las aportaciones de las teorías cognitivo-conductuales para incorporar los principios comunes de los tratamientos avalados de forma empírica, en concreto, reevaluación de las interpretaciones negativas, modificar las conductas desadaptativas, prevenir la evitación emocional y utilizar procedimientos de exposición emocional ante situaciones temidas y volver a activar la conducta en caso de estado de ánimo bajo. [42] Se ha comprobado que estos mecanismos no solo cambian el comportamiento y la experiencia emocional, sino que también producen cambios en las funciones cerebrales. [43]

**Programas para facilitar el acceso a los tratamientos psicológicos**

Atendiendo a la evidencia anterior, se ha convertido en una prioridad facilitar el acceso a los tratamientos psicológicos. Merece la pena destacar la iniciativa que puso en marcha el gobierno del Reino Unido en el año 2007 para ofrecer tratamientos basados en la TCC a los trastornos mentales más comunes en el contexto de AP (principalmente ansiedad y depresión), de acuerdo con las recomendaciones de las guías NICE. [44] El programa se denominó “Mejora del Acceso a las Terapias Psicológicas” (*Improving Access to Psychological Therapies*, IAPT) y ha supuesto el mayor esfuerzo desarrollado hasta ahora a nivel mundial por diseminar los tratamientos psicológicos basados en la evidencia científica a la población general a través de los servicios de AP. Previamente, se desarrollaron dos proyectos piloto durante 13 meses cuyos resultados clínicos cumplieron ampliamente las expectativas. [45,46] Un gran número de pacientes con estos trastornos fueron tratados con intervenciones de baja intensidad (incluyendo distintas modalidades de tratamiento grupal), lo que resulta particularmente útil para lograr una alta rentabilidad. En la evaluación inicial se observó que el 55-56% de los pacientes que habían asistido al menos a dos sesiones, incluyendo la entrevista de evaluación, fueron clasificados como recuperados cuando abandonaron los servicios, y los logros del tratamiento se mantuvieron en gran medida a los 10 meses de seguimiento. En una evaluación posterior, se alcanzaron tamaños del efecto de d=1,39 para los problemas de ansiedad y d=1,41 para los problemas de depresión. Además, se consiguieron altas tasas de recuperación (76% para la depresión y 74% para la ansiedad), la disminución del riesgo de recaída y el mantenimiento de los resultados positivos a largo plazo. [46] De los pacientes que terminaron el curso del tratamiento en IAPT, el 45% se recuperó (basado en un estricto doble criterio, puntuar por debajo del umbral clínico para la ansiedad y la depresión), y un 16% adicional mostró una mejoría poco por debajo de la completa recuperación. [47]

**La situación en España del tratamiento de los tratarnos mentales**

En España no se ha desarrollado una iniciativa similar de diseminación del tratamiento psicológico basado en la evidencia científica, a pesar de que el 65% de las personas con un diagnóstico de trastorno mental en los últimos doce meses no ha recibido tratamiento sanitario alguno en el último año [48] y la carga de las enfermedades mentales es mayor que la de las enfermedades físicas y su inicio precede a éstas. [49] Aunque casi el 60% de las personas con trastorno mental visitan a su médico de AP, el tratamiento psicológico habitualmente no se dispensa en este ámbito, por lo que este tipo de tratamiento solo alcanza a un pequeño porcentaje de pacientes, que es derivado a los Servicios de Atención Especializada (Salud Mental).

Es escasa la proporción de casos diagnosticados con algún trastorno de ansiedad o del estado de ánimo en los últimos doce meses que reciben un tratamiento mínimamente adecuado de acuerdo con la evidencia científica, siendo tan sólo del 31,8% en Atención Especializada (10,8% en el caso del tratamiento psicológico) y 30,5% en AP [50] (los porcentajes más bajos entre los países de nuestro entorno). Del mismo modo, el tratamiento psicológico se aplica sólo al 0,9% de los casos con un trastorno de ansiedad en los últimos doce meses [48], pese a ser el tratamiento de elección [34]; un 27% de estos pacientes recibe tratamiento psicológico y farmacológico, mientras que un 33% recibe tratamiento exclusivamente farmacológico y el 39%, de esa minoría con trastornos mentales que llega a consultar con servicios sanitarios, no recibe ningún tratamiento para este tipo de trastornos. [48]

Distintos factores pueden explicar esta situación, tales como la formación de los profesionales, el tiempo necesario, la sobrecarga asistencial, las actitudes de los profesionales o la organización, o la relación de AP con la atención especializada. Lo cierto es que los tratamientos con apoyo empírico suponen sesiones de al menos 1 hora y con una frecuencia semanal o quincenal, al menos inicialmente. Estas características exceden los recursos que los médicos de AP tienen para atender a sus pacientes. Esto explica la tendencia a utilizar el tratamiento farmacológico como casi única opción terapéutica, incluso en los casos en lo que no es el tratamiento de elección, no es la preferencia del paciente entre los tratamientos disponibles o el paciente no ha respondido favorablemente al mismo en un primer intento. En esta línea, la Estrategia *en* Salud Mental del *Sistema* Nacional *de Salud* reconoce que la marcada tendencia a la sobremedicalización de la salud mental, incluso de los trastornos que deberían tratarse con tratamientos psicológicos. [51]

En conjunto, tenemos una situación en España, y en Cantabria en particular, en la que existe un problema de salud con una alta prevalencia, que ocasiona importantes costes para el afectado, sus allegados y para el conjunto de la sociedad. Sin embargo, pese a la existencia de terapias psicológicas adecuadas y recomendadas por las guías clínicas de referencia como tratamiento de elección para los trastornos mentales más prevalentes, esto es, las basadas en la TCC, éstas apenas se usan en el nivel asistencial por el que se accede al sistema sanitario y en el que se tratan la mayoría de estos problemas de salud: la AP. Pese a que hay algunas iniciativas a nivel mundial para facilitar el acceso a los tratamientos psicológicos, en España no existen estudios rigurosos a nivel metodológico que permitan probar su coste-efectividad en el contexto de AP. Especialmente relevante es el estudio de tratamientos que permitan su aplicación en formatos y enfoques que se puedan diseminar con facilidad por los centros de salud, como es el caso de los tratamientos transdiagnósticos grupales de corta duración.

**Objetivo principal:**

Probar el coste-efectividad y el coste-utilidad del tratamiento cognitivo-conductual transdiagnóstico frente a la terapia de relajación en los Centros de Atención Primaria de Cantabria en el post-tratamiento y en el seguimiento a los 12 meses.

**METODOLOGÍA**

**Descripción del tipo de ensayo**

Se llevará a cabo un ensayo controlado aleatorizado prospectivo, con simple ciego, en dos centros de salud de AP de Cantabria, (Camargo Costa y Camargo Interior). Los pacientes que acuden a su centro de AP con posibles trastornos mentales comunes (relacionados con ansiedad, depresión o somatizaciones) no susceptibles de ser derivados a atención especializada de acuerdo con el criterio de su médico de referencia en el centro de salud, serán invitados a participar en el estudio, así como a firmar el consentimiento informado. Los que acepten serán evaluados antes de ser asignados a los grupos, por lo que los evaluadores serán ciegos a la asignación de tratamiento en todo momento. Los casos que hayan completado las pruebas serán asignados aleatoriamente a las dos condiciones de tratamiento: (i) grupo control con tratamiento mediante técnicas de relajación muscular progresiva de Bernstein y Borkovec junto con el tratamiento habitual por su médico de AP; o (ii) grupo experimental con tratamiento grupal manualizado basado en la TCC más el tratamiento habitual de su médico de AP.

Los pacientes asignados a ambos grupos recibirán 7 sesiones de 1.5 horas de duración, en subgrupos de aproximadamente unas 10-12 personas, repartidas a lo largo de 12 semanas, con una mayor frecuencia temporal en el inicio del tratamiento (una sesión por semana) y progresiva ampliación del intervalo de tiempo entre sesiones al avanzar el tratamiento.

**Tamaño de la muestra**

El estudio reclutará a 300 participantes (150 en cada brazo de tratamiento).

**Nombre de cada brazo**

| Brazo | Intervención asignada |
| --- | --- |
| Experimental: Tratamiento cognitivo-conductual transdiagnóstico (TD-CBT).  Terapia cognitivo-conductual grupal transdiagnóstica: Las intervenciones psicológicas serán manuales. Los pacientes asignados al grupo experimental recibirán 7 sesiones (1,5 h/sesión) en grupos de aproximadamente 8-10 individuos durante un periodo de 12 semanas. | Conductual: Tratamiento cognitivo-conductual transdiagnóstico (TD-CBT). |
| Comparador activo: Relajación muscular  El grupo de control recibirá una intervención grupal de PMR, basada en el procedimiento de Bernstein y Borkoveck. Los pacientes recibirán 7 sesiones (1,5 horas/sesión) en grupos de 8-10 individuos durante un período de 12 semanas. | Relajación muscular progresiva de Bernstein y Borkovec (PMR) |

**Proceso de reclutamiento de los participantes**

En un primer momento, los médicos de AP, tal y como lo hacen habitualmente de entre aquellos pacientes que no derivarían a atención especializada, reclutarán a posibles candidatos con un trastorno mental común (trastornos de ansiedad, depresión leve o moderada, trastornos somatomorfos) como motivo principal de consulta (Fase 1), a continuación, psicólogos formados con las herramientas adecuadas para la detección de los trastornos mentales realizarán un diagnóstico correcto de aquellos trastornos de intensidad leve y moderada que han de tratarse en AP de acuerdo con los criterios de inclusión y de exclusión previamente establecidos (Fase 2). En el caso de que se cumplan los criterios se procederá a la asignación aleatoria a grupos por parte de personal investigador *ciego,* no implicado en la evaluación ni en el tratamiento. En caso de que no cumpla criterios, se descartaría al sujeto, recomendándole la consulta con su médico de AP o, si fuera preciso, derivándolo al Centro de Salud Mental de referencia para su zona.

En la fase de post-tratamiento, un psicólogo que no haya participado en el grupo de tratamiento psicológico realizaría la fase evaluación con las mismas pruebas utilizadas en la evaluación pre-tratamiento. Este investigador sería ciego a la rama del estudio a la que pertenece el sujeto evaluado. Se le pedirá al paciente que no revele en que grupo participó en ningún momento del estudio.

**Consideraciones éticas**

La investigación propuesta respeta y cumplirá la legislación vigente y otras normas reguladoras, pertinentes al proyecto, en materia de ética, incluidos los principios fundamentales de la Declaración de Helsinki, del Convenio del Consejo de Europa relativo a los derechos humanos y la biomedicina, de la Declaración Universal de la UNESCO sobre el genoma humano y los derechos humanos, y del Convenio para la protección de los derechos humanos y la dignidad del ser humano con respecto a las aplicaciones de la Biología y la Medicina.

Este estudio se desarrollará conforme a los siguientes criterios:

- Explicar brevemente los principios éticos que justifican la investigación de acuerdo a una normatividad a nivel internacional y a nivel nacional.
- Expresar claramente los riesgos y las garantías de seguridad que se brindan a los participantes.
- Establecer que la investigación se llevará a cabo cuando se obtenga la autorización: del representante legal de la institución investigadora y de la institución donde se realice la investigación; el Consentimiento Informado de los participantes; y la aprobación del proyecto por parte del Comité de Ética en Investigación de la institución.

**Criterios de inclusión/exclusión:**

Criterios de inclusión:

- Pacientes de 18 a 65 años, ambos inclusive, que acudan al centro de atención primaria en busca de tratamiento por síntomas de ansiedad, depresión o somáticos.

- Puntuaciones superiores a los puntos de corte predeterminados en el GAD-7 (>= 10), el PHQ-9 (>= 10) o el PHQ-15 (>=10 más una puntuación de 2 en tres o más síntomas somáticos).

- Aceptación de participar en el estudio, con consentimiento informado por escrito.

Criterios de exclusión:

- Trastorno depresivo mayor (PHQ-9> 24) y/o discapacidad severa (SDS > 25) serán entrevistados por un clínico por la presencia de cualquier trastorno mental severo, incluyendo trastornos del espectro autista, trastorno bipolar, esquizofrenia, anorexia nerviosa, dependencia de sustancias, trastorno de personalidad.

- Presencia de intentos de suicidio graves o recientes

- Presencia de discapacidad intelectual (CI < 70).

- Estar recibiendo tratamiento psicológico o cualquier tipo de atención especializada relacionada con la salud mental.

- Conocimiento insuficiente del idioma español

**Medidas e instrumentos**

Medidas primarias:

- Cambio en los datos de coste-efectividad [Marco temporal: Línea de base, inmediatamente después de la intervención y 12 meses de seguimiento]

Los resultados de coste-efectividad se calcularán mediante el ICER, definido como la diferencia de costes medios entre las intervenciones dividida por la diferencia de su efectividad según las puntuaciones medias de los cuestionarios de síntomas.

Para el cálculo de los costes se utilizarán los datos de asistencia sanitaria recogidos. Para calcular los costes relacionados con la asistencia sanitaria, se utilizará un cuestionario ad hoc para recoger los datos de asistencia sanitaria relacionados con los trastornos emocionales (consultas sanitarias públicas y privadas, accidentes, pruebas médicas y bajas por enfermedad en los últimos 3 meses; psicofármacos u otra medicación, y su posología).

- Cambio en los datos de coste-utilidad [Marco temporal: Línea de base, inmediatamente después de la intervención, y 12 meses de seguimiento]

La coste-utilidad se medirá a través de los datos sanitarios recogidos anteriormente y de la Escala Europea de Calidad de Vida (EuroQoL, EQ) [71], calculando los QALYS y los ICURs, definidos como la diferencia en el coste medio dividido por la diferencia en los QALYs medios. Se utilizará la versión española del EuroQol de 5 dominios y 5 niveles (EQ-5D-5L) [72, 73] para evaluar el estado de salud en cinco dimensiones (movilidad, autocuidado, actividades diarias, dolor/enfermedad y ansiedad/depresión) con cinco niveles de gravedad (sin problemas, problemas leves, problemas moderados, problemas graves y problemas extremos o incapacidad para realizar la actividad). Las puntuaciones obtenidas en las diferentes dimensiones pueden combinarse para crear un código de 5 dígitos que describa el estado de salud del paciente, pudiendo establecer hasta 3125 combinaciones diferentes, y por tanto, diferentes estados de salud posibles. Este sistema se convirtió en una puntuación de utilidad utilizando el conjunto de valores para España del EQ-5D-5L Crosswalk Index Value descargado de la página web de EuroQol (<https://euroqol.org/euroqol/>).

Medidas secundarias:

- Cambio en los síntomas depresivos: Cuestionario de Salud del Paciente - 9 ítems (PHQ-9) [Marco temporal: Línea de base, inmediatamente después de la intervención y seguimiento a los 12 meses]

El PHQ-9 [54] es el módulo de depresión del PHQ [74,75] que puntúa los 9 criterios de depresión del DSM-IV en las últimas dos semanas. Es una escala de autoinforme de nueve ítems que oscila entre 0 y 27 (las puntuaciones más altas significan un peor resultado).

- Cambio en los síntomas de ansiedad: Trastorno de Ansiedad Generalizada - 7 ítems (GAD-7) [Marco temporal: Línea de base, inmediatamente después de la intervención, y 12 meses de seguimiento]

El GAD-7 [56] evalúa los síntomas comunes de ansiedad en las últimas dos semanas. Se compone de siete ítems de autoinforme que van de 0 a 21 puntos. Las puntuaciones más altas significan una mayor presencia de síntomas de ansiedad.

- Cambio en los síntomas somáticos: Cuestionario de Salud del Paciente - 15 ítems (PHQ-15) [Marco temporal: Línea de base, inmediatamente después de la intervención, y 12 meses de seguimiento]

El PHQ-15 [58] es el módulo de somatización del PHQ y puntúa los síntomas presentes en las últimas cuatro semanas. La escala está compuesta por quince ítems de autoinforme, que van de 0 a 30. Las puntuaciones más altas significan un peor resultado.

- Cambio en el funcionamiento: Escala de discapacidad de Sheehan (SDS) [Marco temporal: Línea de base, inmediatamente después de la intervención, y 12 meses de seguimiento]

La SDS [59] es una escala autoinformada de cinco ítems compuesta por tres dominios principales (trabajo, familia y funcionamiento social) y dos ítems opcionales (estrés percibido y apoyo social percibido). Va de 0 a 50, y las puntuaciones más altas indican un peor resultado.

- Cambio en la satisfacción con el tratamiento [Marco temporal: Inmediatamente después de la intervención y a los 12 meses de seguimiento]

Las evaluaciones posteriores al tratamiento y al seguimiento de 12 meses también recogerán una pregunta adicional sobre la satisfacción con el tratamiento, mediante una pregunta tipo Likert, que va de 0 a 10.

Otras medidas de resultado mecanicistas:

- Cambio en la rumiación: Escalas de respuestas rumiantes (subescala de rumiación) (RRS-B) [Marco temporal: Línea de base, inmediatamente después de la intervención y seguimiento de 12 meses]

La RRS-B [76] está compuesta por cinco ítems autoinformados, que van de 5 a 20. Las puntuaciones más altas significan un peor resultado.

- Cambio en la preocupación: Penn State Worry Questionnaire - Abbreviated (PSWQ-A) [Marco temporal: Línea de base, inmediatamente después de la intervención, y 12 meses de seguimiento]

El PSWQ-A [62] mide la preocupación patológica como un estado incontrolable y general. La escala está compuesta por ocho ítems autoinformados, que van de 5 a 40. Las puntuaciones más altas significan un peor resultado.

- Cambio en los sesgos atencionales e interpretativos: Inventario de Actividad Cognitiva en Trastornos de Ansiedad (IACTA) [Marco temporal: Línea de base, inmediatamente después de la intervención y seguimiento de 12 meses]

El IACTA fue desarrollado originalmente por Cano-Vindel [63]. Incluye subescalas que evalúan las distorsiones según la teoría de los cuatro factores de Eysenck [77]. La escala está compuesta por cinco ítems autoinformados, que van de 0 a 20. Las puntuaciones más altas significan un peor resultado.

- Cambio en la regulación de las emociones: Cuestionario de regulación cognitiva de las emociones (CERQ) [Marco temporal: Línea de base, inmediatamente después de la intervención y seguimiento a los 12 meses]

El CERQ-36 [78] se desarrolló para medir las estrategias específicas de regulación cognitiva de las emociones que una persona utiliza para afrontar un acontecimiento estresante (autoculpabilización, aceptación, rumiación, reenfoque positivo, reenfoque en la planificación, reevaluación positiva, puesta en perspectiva, catastrofización o culpabilización de los demás). Puntúa de 1 ("casi nunca") a 5 ("casi siempre") la frecuencia con la que el participante piensa como se describe. Se utilizará la versión abreviada de 27 ítems [79]. Cada estrategia cognitiva se evalúa mediante tres ítems, que van de 3 a 15. Las puntuaciones más altas significan un mayor uso de la estrategia.

- Cambio en las creencias metacognitivas: Cuestionario de Metacogniciones (subescala de creencias negativas) (MCQ) [Marco temporal: Línea de base, inmediatamente después de la intervención y seguimiento a los 12 meses]

El MCQ-NB [65] es una forma corta del MCQ original [80], que mide las creencias sobre los propios procesos de pensamiento. La escala está compuesta por seis ítems autoinformados, que van de 6 a 24. Las puntuaciones más altas significan un peor resultado.

- Cambio en las distorsiones cognitivas en los trastornos emocionales (CDTE) [Marco temporal: Línea de base, inmediatamente después de la intervención, y 12 meses de seguimiento]

El CDTE [81] mide la frecuencia de ciertos sesgos cognitivos. Incluye dieciséis ítems autoinformados que miden la presencia de cuatro factores: sesgo de atención sostenida, sesgo de atención dividida, sesgo interpretativo de magnificación y sesgo interpretativo de catastrofización. Va de 0 a 4. Las puntuaciones más altas significan una mayor presencia del sesgo cognitivo.

- Cambio en la alianza: Working Alliance Inventory Patient Form (WAI-P) y Group Session Rating Scale (GSRS) [Marco tamporal: En las sesiones de terapia número 1, 4 y 7].

El WAI-P [69] es una escala de autoinforme de treinta y seis que mide la alianza terapéutica percibida. Oscila entre 36 y 252 y las puntuaciones más altas indican una mejor alianza entre el paciente y el profesional clínico.

La GSRS [70] es una escala de autoinforme de cuatro ítems que evalúa la alianza con el grupo. Va de 0 a 40, y las puntuaciones más altas indican una mejor alianza entre el paciente y el grupo de terapia.

**El tratamiento psicológico transdiagnóstico, grupal del grupo experimental**

La intervención psicológica ofertada (que será aplicada por psicólogos) estará protocolizada (con un Manual del Terapeuta) y descrita en un documento dirigido al paciente. Los pacientes asignados al grupo experimental recibirán 7 sesiones de 1.5 horas de duración en grupos de aproximadamente unas 10 personas, repartidas a lo largo de 24 semanas, con una mayor frecuencia temporal en el inicio del tratamiento (una sesión por semana) y progresiva ampliación del intervalo de tiempo entre sesiones al avanzar el tratamiento. El protocolo del tratamiento se ajustará a los siguientes contenidos:

*Sesiones 1*: Este grupo iniciará el tratamiento psicológico con un entrenamiento psicoeducativo, con el fin de dotar a los participantes de información concreta (oral y escrita), así como recursos de información (e.g., libros, página Web), sobre el estrés, las emociones, la ansiedad, la ira, la depresión, el papel de los procesos y sesgos cognitivos sobre la emoción, el aprendizaje emocional, la regulación emocional, las relaciones entre emoción y conducta, etc., procurando adaptar los contenidos a las necesidades de los participantes. En todas las sesiones se motivará a los pacientes para tomar un papel activo en el tratamiento y desarrollar las tareas para casa diarias correspondientes.

*Sesión 2*: Se introducirán las técnicas de relajación, con una mezcla de entrenamiento en relajación muscular progresiva de Bernstein y Borkovec, en respiración abdominal y en imaginación. Se explicarán los principios de estos ejercicios, los objetivos que se pretenden y la forma de realizar el entrenamiento. Además, se pedirá a los pacientes la práctica diaria en casa de estos ejercicios con una grabación de audio que se les suministrará, con una duración aproximada de unos 30 minutos. Para el desarrollo de la motivación y el hábito de la práctica de esta tarea se pedirá la realización de un autorregistro gráfico sobre la valoración diaria del grado de relajación experimentado tras la práctica, a lo largo de las 24 semanas de intervención.

*Sesiones 3-4*: Se dedicarán a la realización de práctica en reestructuración cognitiva y entrenamiento en autorregulación emocional. [66, 67] Primero se les instruirá, de manera general, en qué son las emociones, cómo se manifiestan (i.e., a nivel cognitivo-subjetivo, fisiológico y conductual) la ansiedad, el miedo o la tristeza; así como en qué situaciones se producen estas respuestas, qué reacciones ayudan a mejorar la adaptación al entorno o cuáles no, y qué estrategias son adecuadas y cuáles no para regular dichas reacciones. También se explicará cómo se pueden desarrollar fácilmente algunos trastornos emocionales (e.g., cuando aumenta el estrés, se desatan reacciones descontroladas de ansiedad, se da mucha importancia a una respuesta fisiológica que no presenta control voluntario y se focaliza la atención en las sensaciones físicas que produce). Con exposición de casos, se les mostrará cómo es posible la detección y cambio de pensamientos deformados, creencias irracionales, valoraciones amenazantes [68], sesgos cognitivos [52], estrategias de regulación emocional, etc., que están asociados con estados emocionales negativos intensos de ansiedad, ira u otras emociones negativas en la vida cotidiana. Después, se trabajará con muestras concretas de sucesos emocionales vividos por los pacientes en la última semana y se les mostrará cómo deben enfocar dichos sucesos desde una nueva perspectiva cognitivo-emocional que les ayudará entender mejor sus errores y sesgos en la regulación emocional, y cómo pueden restaurar esa autorregulación emocional mediante cambios en el procesamiento de la información (e.g., fomentar el procesamiento de información neutra o positiva, utilizar la distracción, reducir la importancia y magnificación de la amenaza o la pérdida, reducir los procesos rumiativos, la preocupación y sesgos de la atención centrada en la amenaza o la pérdida, aumentar las autoinstrucciones positivas y la autoeficacia percibida, etc.).

*Sesiones 5-6*: Se centrarán en las técnicas conductuales e irán dirigidas a que los pacientes aprendan o reaprendan y vuelvan a recuperar en su vida cotidiana el autocontrol de situaciones, emociones y conductas que habían dejado de manejar correctamente. [66,67] Esta intervención debe incluir un paquete de tratamiento compuesto por varias técnicas psicológicas (e.g., la exposición) que se inician en las sesiones grupales y deben practicar diariamente en casa (además de relajación y libro de autoayuda): autoobservación, control de estímulos, entrenamiento conductual reforzado, exposición sin conductas de seguridad, entrenamiento en habilidades de afrontamiento, etc. En las sesiones grupales se revisarán los sucesos emocionales vividos recientemente, se refuerzan los logros, se corrigen errores y se sigue animando a disminuir los sesgos cognitivos (e.g., atención, interpretación, memoria, atribución) que desatan las reacciones emocionales que alteran la conducta, poniendo más tareas para casa que implican ensayo, práctica, refuerzo, corrección, exposición progresiva, etc.

*Sesión 7*: Incluirá la prevención de recaídas, señalando que éstas no significan una vuelta al principio, una pérdida total de las ganancias terapéuticas, ni un fracaso, sino un nuevo reto que hay que superar y una oportunidad para aprender más y consolidar lo aprendido. Para ello, se deben analizar las recaídas que puedan haber acontecido, señalando cómo se debe proceder para superar las dificultades y aprender de tales tropiezos. Se completará la sesión con un repaso general de todo lo aprendido y una generalización a problemas similares que puedan surgir en el futuro, remarcando siempre que para superar dichas dificultades se poseen ahora unas herramientas técnicas, así como nuevas habilidades de afrontamiento (que se continúan desarrollando a través de la práctica con dichas técnicas), las cuales se han mostrado útiles para superar los problemas que ya se han resuelto. Se recomendará que para afrontar el estrés vital futuro se recurra a estas habilidades y no a los viejos hábitos que derivaron en el desarrollo de sus problemas emocionales.

**Tratamiento mediante técnicas de relajación**

El tratamiento mediante la técnica de relajación muscular progresiva de Bernstein y Borkovec será aplicado por psicólogos. Los pacientes asignados al grupo control, al igual que en el grupo experimental, recibirán 7 sesiones de 1.5 horas de duración en grupos de aproximadamente unas 10 personas, repartidas a lo largo de 24 semanas, con una mayor frecuencia temporal en el inicio del tratamiento (una sesión por semana) y progresiva ampliación del intervalo de tiempo entre sesiones al avanzar el tratamiento.

Ambos grupos, el experimental y el control, junto con los tratamientos anteriormente descritos, seguirán el tratamiento habitual por su médico de AP, según su procedimiento habitual, típicamente tratamiento farmacológico y apoyo no estructurado. Se tendrá en cuenta el número de consultas recibidas durante el periodo de participación en el estudio.

**Análisis estadístico**

Se comprobará la equivalencia de ambos grupos paralelos en las medidas antes del tratamiento. En el análisis de la eficacia, se llevarán a cabo ANOVAs intergrupo-intragrupo 2x2 (grupo x tratamiento) en las medidas dependientes: 1) síntomas de ansiedad, depresión y somatizaciones; 2) factores cognitivo-emocionales asociados a los síntomas; 3) disfunción laboral, familiar y social; 4) calidad de vida; 5) satisfacción con la atención recibida; 6) análisis de coste-eficacia (e.g., frecuentación, consumo de fármacos). Se calculará el tamaño del efecto de ambos grupos sobre las distintas variables dependientes, así como su precisión (intervalos de confianza al 95%), teniendo en cuenta el número de sesiones recibidas. Se calcularán también los porcentajes de pacientes de ambos grupos que han disminuido en un 50% y en una desviación típica sus puntuaciones en síntomas clínicos, así como los porcentajes de probables casos con trastornos mentales (de ansiedad, depresivos y somatomorfos) antes y después del tratamiento, según los puntos de corte y reglas de decisión para el diagnóstico. Los análisis primarios seguirán el principio de “intención de tratar”, de manera que todos los individuos que han ingresado en el estudio serán considerados en su grupo correspondiente, aunque no hayan cumplido con su protocolo. Esto permitirá mantener hasta el final del estudio el objetivo de la aleatorización, es decir, disminuir la probabilidad de sesgar los resultados.

El análisis coste-efectividad se realizará calculando el incremento de los ratios de coste-efectividad, definidos por la diferencia de la media de los costes dividida entre el incremento de la efectividad entre las diferentes alternativas terapéuticas comparadas (ICER).

**CRONOGRAMA Y PLAN DE TRABAJO**

*Meses 1*: Implicación de profesionales en el proyecto y entrenamiento de los psicólogos clínicos en el tratamiento psicológico grupal mediante un taller conducido por expertos de la Sociedad Española para el Estudio de la Ansiedad y el Estrés (SEAS), bajo supervisión del Prof. Antonio Cano Vindel.

*Meses 2-11*: Reclutamiento de pacientes (progresivo durante 10 meses) por parte de los médicos de AP en los Centros de Salud, aleatorización, realización de los grupos de terapia y evaluaciones por parte de los psicólogos clínicos, recogida de datos.

*Meses 10-12*: Se completa la recogida y análisis de datos y se generaran los borradores de los artículos. Divulgación de datos preliminares del proyecto en congresos nacionales e internacionales.

**APLICABILIDAD Y UTILIDAD DE LOS RESULTADOS Y CAPACIDAD PARA SER PROTEGIDOS Y TRANSFERIDOS AL MERCADO**

El proyectado ensayo clínico proporcionará una evidencia clara sobre que, en comparación con el tratamiento a través de la relajación, la incorporación de un tratamiento grupal para los trastornos mentales comúnmente tratados en los Centros de Salud ayuda a reducir la sintomatología ansiosa, depresiva y somática, así como una reducción de los costes (directos e indirectos) asociados a los trastornos de ansiedad, del estado de ánimo y somatomorfos (Hipótesis principal). El PsicAP-Costs2 permite una transferencia inmediata de los resultados al contexto clínico al proporcionar la primera experiencia en nuestro contexto de un tratamiento grupal transdiagnóstico manualizado de corta duración (7 sesiones), que lo hace fácilmente aplicable y generalizable a los centros de salud de Cantabria y del resto del SNS. El beneficio económico esperado en la reducción de los costes del tratamiento psicofarmacológico y por medio de la disminución del gasto en bajas laborales y pérdida de productividad, que es un objetivo prioritario del SCS en el manejo de las IT asociadas a trastornos mentales comunes (Plan de Salud Mental de Cantabria 2015-2019), deben hacer rentable la diseminación entre los profesionales sanitarios de este tipo de tratamiento protocolizado, de bajo coste y de corta duración, fácilmente integrable en la asistencia rutinaria ofrecida en los Centros de Salud.

**Ubicaciones**

España

Centro Sanitario "Camargo Costa" Reclutando

C. Julio de Pablo, 0, 39600, Maliaño, Cantabria

Centro Sanitario "Camargo Interior" Reclutando

Av. de Bilbao, s/n, 39600 Maliaño, Cantabria

Centro Sanitario "Sardinero" Reclutando

C. Alcalde Vega Lamera, 6, 39005 Santander, Cantabria

Centro Sanitario "Dávila" Reclutando

P.º del Gral. Dávila, 71, 39006 Santander, Cantabria

**Patrocinadores y colaboradores**

Instituto de Investigación Sanitaria Valdecilla (IDIVAL)

Agencia Estatal de Investigación

**BIBLIOGRAFÍA**

1. Haro JM, Palacín C, Vilagut G, Martínez M, Bernal M, Luque I, Codony M, DolzM, Alonso J; Grupo ESEMeD-España. Prevalencia de los trastornos mentales y factores asociados: resultados del estudio ESEMeD-España. Med Clin (Barc). 2006 Apr 1;126(12):445-51.

2. Vázquez-Barquero JL, Díez-Manrique JF, Peña C, Aldama J, Samaniego Rodríguez C, Menéndez Arango J, Mirapeix C. A community mental health survey in Cantabria: a general description of morbidity. Psychol Med. 1987 Feb;17(1):227-41.

3. Haro JM, Pinto-Meza A, Serrano-Blanco A. Epidemiología de los trastornos mentales en Atención Primaria. En Psiquiatría en Atención Primaria. José Luis Vázquez-Barquero (Ed.). Madrid: Grupo Aula Médica, S.L. 2007:41-59.

4. Layard R, Clark DM. Thrive: The Power of Evidence-Based Psychological Therapies. London: Penguin. 2014.

5. Wittchen HU, Jacobi F, Rehm J, Gustavsson A, Svensson M, Jönson B, Olesen J, Allgulander C, Alonso J, Faravelli C et al. The size and burden of mental disorders and other disorders of the brain in Europe 2010. European Neuropsychopharmacology. 2011;21:655-679.

6. Gobierno de Cantabria. Plan de Salud Mental de Cantabria 2014-2019. Santander: Consejería de Sanidad y Servicios Sociales. 2014.

7. Calvo Bonacho E. Duración de la incapacidad temporal asociada a diferentes patologías en trabajadores españoles: discusión acerca de la ITCC en los trastornos mentales. Madrid. 2010. Disponible en http://www.seg-social.es/prdi00/groups/public/documents/binario/146666.pdf. Fecha acceso: 8.02.2016.

8. Gustavsson A, Svensson M, Jacobi F, Allgulander C, Alonso J, Beghi E, Dodel R et al. Cost of disorders of the brain in Europe 2010. European Neuropsychopharmacology. 2011;21:718-779.

9. Parés-Badell O, Barbaglia G, Jerinic P, Gustavsson A, Salvador-Carulla L, Alonso J. Cost of disorders of the brain in Spain. PLoS One.2014;18:9(8):e105471.

10. Melek S, Norris D. Chronic Conditions and Comorbid Psychological Disorders. Seattle: Milliman. 2008.

11. De Hert M, Correll CU, Bobes J, Cetkovich-Bakmas M, Cohen D, Asai I, Detraux J, Gautam S, Möller HJ, Ndetei DM, Newcomer JW, Uwakwe R, Leucht S. Physical illness in patients with severe mental disorders. I. Prevalence, impact of medications and disparities in health care. World Psychiatry.2011;10(1):52-77.

12. Zuidersma M, Ormel J, Conradi HJ, de Jonge P. An increase in depressive symptoms after myocardial infarction predicts new cardiac events irrespective of depressive symptoms before myocardial infarction. Psychol Med.2012;42:683-93.

13. Joukamaa M, Heliövaara M, Knekt P, Aromaa A, Raitasalo R, Lehtinen V. Mental disorders and cause-specific mortality. Br J Psychiatry. 2001 Dec;179:498-502.

14. Nordentoft M, Wahlbeck K, Hällgren J, Westman J, Osby U, Alinaghizadeh H, Gissler M, Laursen TM. Excess mortality, causes of death and life expectancy in 270,770 patients with recent onset of mental disorders in Denmark, Finland and Sweden. PLoS One.2013; 8(1):e55176.

15. Cuijpers P, Smit F. Excess mortality in depression: a meta-analysis of community studies. J Affect Disord. 2002 Dec;72(3):227-36.

16. Harris EC, Barraclough B. Suicide as an outcome for mental disorders. A meta-analysis. Br J Psychiatry.1997 Mar; 170: 205-28.

17. Arsenault-Lapierre G, Kim C, Turecki G. Psychiatric diagnoses and 3275 suicides: a metanalysis. BMC Psychiatry.2004;4:37.

18. Instituto Nacional de Estadística. Defunciones según la causa de muerte Madrid: Instituto Nacional de Estadística. 2013 [Documento Internet, Acceso 08/02/2016]. Disponible en: www.ine.es.

19. Luoma JB, Martin CE, Pearson JL. Contact with mental health and primary care providers before suicide: a review of the evidence. Am J Psychiatry. 2002;159:909-16.

20. Wittchen H.-U, Jacobi F. Size and burden of mental disorders in Europe_a critical review and appraisal of 27 studies. Eur. Neuropsychopharmacol. 2005;15(4):357-376.

21. Kessler RC, Ustun B. The WHO World Mental Health Surveys: Global Perspectives on the Epidemiology of Mental Disorders. New York: Cambridge University Press .2008.

22. Butler AC, Chapman JE, Forman EM, Beck AT. The empirical status of cognitive-behavioral therapy: a review of meta-analyses. Clin Psychol Rev. 2006;26:17-31.

23. Cuijpers P, Berking M, Andersson G, Quigley L, Kleiboer A, Dobson KS. A meta-analysis of cognitive-behavioural therapy for adult depression, alone and in comparison with other treatments. Can J Psychiatry. 2013 Jul;58(7):376-85.

24. Hollon SD, Stewart MO, Strunk DR. Enduring effects for cognitive behaviour therapy in the treatment of depression and anxiety. Annu Rev Psychol. 2006;57:285–315.

25. Spielmans GI, Berman MI, Usitalo AN. Psychotherapy versus second-generation antidepressants in the treatment of depression: a meta-analysis. J Nerv Ment Dis. 2011;199:142–9.

26. Van Orden M, Hoffman T, Haffmans J, Spinhoven P, YHoencamp E. Collaborative Mental Health Care versus Care as Usual in a Primary Care Setting: a randomised controlled trial. Psychiatr Serv. 2009;60(1):74-9.

27. Twomey C, O'Reilly G, Byrne M. Effectiveness of cognitive behavioural therapy for anxiety and depression in primary care: a meta-analysis. Fam Pract. 2015 Feb;32(1):3-15.

28. Linde et al. Effectiveness of Psychological Treatments for Depressive Disorders in Primary Care: Systematic Review and Meta-Analysis. 2015.

29. Feng C, Chu H, Chen C, Chang Y, Chang Y, Chen T, . . . & Cou K. The effect of cognitive behavioral group therapy for depression: A metaanalysis 2000-2010. World Views on Evidence-Based Nursing. 2011; First Quarter: 2-16.

30. Huntley A, Araya R, Salisbury C. Group psychological therapies for depression in the community: Systematic review and meta-analysis. The British Journal of Psychiatry. 2012; 200: 184-190.

31. González González S, Fernández Rodríguez C, Pérez Rodríguez J, Amigo I. Prevención secundaria de la depresión en atención primaria. Psicothema. 2006 Aug;18(3):471-7.

32. Segarra, G., Farriols, N., Palma, S., Segura, J., & Castell, R. (). Tratamiento psicológico grupal para los trastornos de ansiedad en el ámbito de la salud pública. Ansiedad & Estrés. 2011;17,185-197.

33. National Institute for Health and Clinical Excellence (NICE). Common Mental Health Disorders: Identification and Pathways to Care. NICE Clinical Guideline 123. 2011. Accesible en www.nice.org.uk/CG123 [NICE guideline].

34. McHugh RK, Whitton SW, Peckham AD, Welge JA, Otto MW. Patient preference for psychological vs pharmacologic treatment of psychiatric disorders: a meta-analytic review. J Clin Psychiatry. 2013 Jun; 74(6): 595-602.

35. Swift JK, Greenberg RP, Tompkins KA, Parkin SR. Treatment refusal and premature termination in psychotherapy, pharmacotherapy, and their combination: A meta-analysis of head-to-head comparisons. Psychotherapy (Chic). 2017;54:47-57.

36. Swift JK, Callahan JL, Vollmer BM. Preferences. J Clin Psychol. 2011 Feb; 67(2):155-65.

37. Mergl R, Henkel V, Allgaier AK, Kramer D, Hautzinger M, Kohnen R, Coyne J,Hegerl U. Are treatment preferences relevant in response to serotonergic antidepressants and cognitive-behavioral therapy in depressed primary care patients? Results from a randomized controlled trial including a patients' choice arm. Psychother Psychosom. 2011; 80(1): 39-47.

38. Autonell J, Vila F, Pinto-Meza A, Vilagut G, Codony M, Almansa J, Muñoz PE, Torres JV, Alonso J, Haro JM. Prevalencia-año de la comorbilidad de los trastornos mentales y factores de riesgo sociodemográficos asociados en la población general de España. Resultados del estudio ESEMeD-España. Actas Esp Psiquiatr. 2007; 35 Suppl 2:4-11.

39. Cordero-Andrés, P., González-Blanch, C., Umaran-Alfageme, O., Muñoz-Navarro, R., Ruíz-Rodríguez, P., Medrano, L.A., et al. Tratamiento psicológico de los trastornos emocionales en atención primaria: fundamentos teóricos y empíricos del estudio PsicAP. Ansiedad y Estrés, 2017; 23: 91-98.

40. Brown TA, Barlow DH. A proposal for a dimensional classification system based on the shared features of the DSM-IV anxiety and mood disorders: implications for assessment and treatment. Psychol Assess. 2009 Sep;21(3):256-71.

41. Tortella-Feliu M, Aguayo B, Sesé A, Morillas-Romero A, Balle M, Gelabert JM, Bornas X, Llabrés J. Effects of temperament and emotion regulation styles in determining negative emotional states. Actas Esp Psiquiatr. 2012; 40(6):315-22.

42. Barlow, D. H., Farchione, T. J., Fairholme, C. P., Ellard, K. K., Boisseau, C. L., Allen, L. B., Ehrenreich-May, J. (2015) Protocolo unificado para el tratamiento transdiagnóstico de los trastornos emocionales. Madrid: Alianza Editorial.

43. Porto PR, Oliveira L, Mari J, Volchan E, Figueira I, Ventura P. Does cognitive behavioral therapy change the brain? A systematic review of neuroimaging in anxiety disorders. J Neuropsychiatry Clin Neurosci. 2009;21(2):114-25.

44. National Institute for Health and Clinical Excellence (NICE). Putting guidance into practice. In. UK: NICE. Acceso 22-July-2011, de http://www.nice.org.uk/usingguidance/using_guidance.jsp; 2010.

45. Clark DM, Layard R, Smithies R, Richards DA, Suckling R, Wright B. Improving access to psychological therapy: Initial evaluation of two UK demonstration sites. Behav Res Ther. 2009; 47(11): 910-20.

46. Richards DA, Suckling R. Improving access to psychological therapies: phase IV prospective cohort study. Br J Clin Psychol. 2009; 48(Pt 4): 377-96.

47. Health and Social Care Information Centre Psychological Therapies. Annual Report on the Use of IAPT Services: England. HSCIC. 2014.

48. Codony M, Alonso J, Almansa J, Vilagut G, Domingo A, Pinto-Meza A, et al. Utilización de los servicios de salud mental en la población general española. Resultados del estudio ESEMeD-Espana. [Mental health care use in the Spanish general populations. Results of the ESEMeD-Spain study]. Actas Esp Psiquiatr. 2007; 35 (Suppl 2): 21-8.

49. Kessler RC, Aguilar-Gaxiola S, Alonso J, Chatterji S, Lee S, Ormel J, et al. The global burden of mental disorders: an update from the WHO World Mental Health (WMH) surveys. Epidemiol Psichiatr Soc. 2009; 18(1):23-33.

50. Fernández A, Haro JM, Codony M, Vilagut G, Martinez-Alonso M, Autonell J, et al. Treatment adequacy of anxiety and depressive disorders: primary versus specialised care in Spain. J Affect Disord. 2006; 96(1-2):9-20.

51. SNS – Sistema Nacional de Salud. Estrategia en Salud Mental, 2011. [Accesso 13/02/2016] http://www.msssi.gob.es/organizacion/sns/planCalidadSNS/docs/saludmental/SaludMental2009-2013.pdf.

52. Eysenck MW, Derakshan N. Un marco teórico cognitivo para los trastornos de ansiedad. Ansiedad y Estrés. 1997; 3(2-3):121-134.

53. Kroenke K, Spitzer RL, Williams JB, Lowe B. The Patient Health Questionnaire Somatic, Anxiety, and Depressive Symptom Scales: a systematic review. Gen Hosp Psychiatry. 2010;32(4):345-59.

54. Kroenke K, Spitzer RL, Williams JB. The PHQ-9: validity of a brief depression severity measure. J Gen Intern Med. 2001;16(9):606-13.

55. Kroenke K, Spitzer RL, Williams JB, Lowe B. An ultra-brief screening scale for anxiety and depression: the PHQ-4. Psychosomatics. 2009;50(6):613-21.

56. Spitzer RL, Kroenke K, Williams JB, Lowe B. A brief measure for assessing generalized anxiety disorder: the GAD-7. Arch Intern Med. 2006; 166(10): 1092-7.

57. Wittkampf KA, Baas KD, van Weert HC, Lucassen P, Schene AH. The psychometric properties of the panic disorder module of the Patient Health Questionnaire (PHQ-PD) in high-risk groups in primary care. J Affect Disord. 2011; 130(1-2): 260-7.

58. Kroenke K, Spitzer RL, Williams JB. The PHQ-15: validity of a new measure for evaluating the severity of somatic symptoms. Psychosom Med. 2002; 64(2): 258-66.

59. Sheehan DV, Harnett-Sheehan K, Raj BA. The measurement of disability. Int Clin Psychopharmacol. 1996; 11(Suppl 3): 89-95.

60. Rocha NS, Power MJ, Bushnell DM, Fleck MP. Cross-Cultural Evaluation of the WHOQOL-BREF Domains in Primary Care Depressed Patients Using Rasch Analysis. Med Decis Making. 2012; 32(1): 41-55.

61. Nolen-Hoeksema S, Morrow J. A prospective study of depression and posttraumatic stress symptoms after a natural disaster: the 1989 Loma Prieta Earthquake. J Pers Soc Psychol. 1991; 61(1): 115-21.

62. Meyer TJ, Miller ML, Metzger RL, Borkovec TD. Development and validation of the Penn State Worry Questionnaire. Behav Res Ther. 1990; 28(6): 487-95.

63. Cano-Vindel A. Inventario de Actividad Cognitiva en los Trastornos de Ansiedad (IACTA). In: Facultad de Psicología. Universidad Complutense de Madrid. No publicado. Madrid. 2001.

64. Dominguez-Lara, S., & Medrano, L. (2016a). Propiedades psicométricas del Cognitive Emotional Regulation Questionnaire (CERQ) en estudiantes universitarios de Lima. Psychologia: Avances en la disciplina, 10(1), 53 – 67.

65. Wells A, Cartwright-Hatton S. A short form of the metacognitions questionnaire: properties of the MCQ-30. Behav Res Ther. 2004; 42(4): 385-96.

66. Cano-Vindel A. Bases teóricas y apoyo empírico de la intervención psicológica sobre los desórdenes emocionales en Atención Primaria. Una actualización. Ansiedad y Estrés. 2011; 17(2-3): 157-184.

67. Cano-Vindel A, Dongil-Collado E, Salguero JM, Wood CM. Intervención cognitivo-conductual en los trastornos de ansiedad: una actualización. Informació Psicològica. 2011; 102: 4-27.

68. Cano-Vindel A. Técnicas cognitivas en el control del estrés. In: Fernández-Abascal EG, Jiménez Sánchez MP, editores. Control del Estrés. Madrid: UNED Ediciones. 2002: 247-271.

69. Andrade-González N, Fernández-Liria A. (2015). Spanish adaptation of the Working Alliance Inventory (WAI): Psychometric properties of the patient and therapist forms (WAI-P and WAI-T). *Anales de Psicología, 31*, 524–533.

70. Duncan, B. L., & Miller, S. D. (2007). The group session rating scale. Jenson Beach, FL: Author.

71. EuroQol Group. EuroQol--a new facility for the measurement of health-related quality of life. Health Policy. 1990 Dec;16(3):199-208.

72. Badia X, Roset M, Montserrat S, Herdman M, Segura A. [The Spanish version of EuroQol: a description and its applications. European Quality of Life scale]. Med Clin (Barc). 1999;112 Suppl 1:79-85. Review. Spanish.

73. van Reenen M, Janssen B, Stolk E, Secnik Boye K, Herdman M, Kennedy-Martin M, et al. EQ-5D-5L User Guide. 3.0. EuroQol Research Foundation; 2019. Available from: <https://euroqol.org/publications/user-guides/>

74. Diez-Quevedo C, Rangil T, Sanchez-Planell L, Kroenke K, Spitzer RL. Validation and utility of the patient health questionnaire in diagnosing mental disorders in 1003 general hospital Spanish inpatients. Psychosom Med. 2001 Jul-Aug;63(4):679-86.

75. Spitzer RL, Kroenke K, Williams JB. Validation and utility of a self-report version of PRIME-MD: the PHQ primary care study. Primary Care Evaluation of Mental Disorders. Patient Health Questionnaire. JAMA. 1999 Nov 10;282(18):1737-44

76. Nolen-Hoeksema S, Morrow J. A prospective study of depression and posttraumatic stress symptoms after a natural disaster: the 1989 Loma Prieta Earthquake. J Pers Soc Psychol. 1991 Jul;61(1):115-21.

77. Eysenck MW. A cognitive approach to trait anxiety. Eur J Pers. 2000 Sep;14(5):463-76

78. Garnefski N, Kraaij V, Spinhoven P. Negative life events, cognitive emotion regulation and emotional problems. Pers Individ Dif. 2001;30(8):1311-27.

79. Holgado-Tello, F. P., Amor, P. J., Lasa-Aristu, A., Domínguez-Sánchez, F. J., & Delgado, B. J. A. D. P. A. o. P. (2018). Two new brief versions of the Cognitive Emotion Regulation Questionnaire and its relationships with depression and anxiety. 34(3), 458-464.

80. Cartwright-Hatton S, Wells A. Beliefs about worry and intrusions: the Meta-Cognitions Questionnaire and its correlates. J Anxiety Disord. 1997 May-Jun;11(3):279-96.

81. The PsicAP Group. Cuestionario de Distorsiones Cognitivas en Trastornos Emocionales (CDTE) [Questionnaire of Cognitive Distortions in Emotional Disorders (CDTE)]. Unpublished
